# Interactions between gut microbiota and metabolites modulate cytokine network imbalances in women with unexplained miscarriage

**DOI:** 10.1038/s41522-021-00199-3

**Published:** 2021-03-17

**Authors:** Yongjie Liu, Hainan Chen, Liping Feng, Jun Zhang

**Affiliations:** 1 Ministry of Education and Shanghai Key Laboratory of Children’s Environmental Health, Xinhua Hospital, Shanghai Jiao Tong University School of Medicine, Shanghai, China; 2 Department of Obstetrics and Gynecology, Xinhua Hospital, Shanghai Jiao Tong University School of Medicine, Shanghai, China; 3 Department of Obstetrics and Gynecology, Duke University School of Medicine, Durham, USA

**Keywords:** Clinical microbiology, Microbiome

## Abstract

A dysregulation of cytokine networks has been suggested to be involved in the pathogenesis of unexplained pregnancy loss. Gut microbiota affects host immune response and induces an imbalance in cytokine levels. However, how gut microbial dysbiosis disturbs cellular immune function in miscarriage remains inconclusive. Here we report that IL-2, IL-17A, IL-17F, TNF-α, and IFN-γ are significantly increased in serum of miscarriage patients. Fecal microbiome analyses indicate that microbial diversity and the relative abundances of *Prevotella_1*, *Prevotellaceae_UCG_003* and *Selenomonas_1* are significantly reduced in the cases. Correlation analyses indicate that some microbe-associated metabolites are positively associated with changes in levels of Th1/Th17 cytokines in the miscarriage group. Moreover, we identify that imidazolepropionic acid and 1,4-methylimidazoleacetic acid are associated with subsequent recurrent miscarriage. Our study highlights the network among gut microbiota, fecal metabolites and Th1/Th17-mediated immune response in miscarriage patients and explores the potential predictive values of two fecal metabolites for recurrent miscarriages.

## Introduction

Miscarriage is the most common complication during pregnancy and affects ~15% of clinically recognized pregnancies^1^. After ruling out chromosomal, endocrine, infectious, anatomic, and autoimmune factors, more than 50% of miscarriage cases remain unexplained^2^. In recent years, immune cell dysfunction has been shown to be a risk factor for the pathogenesis of unexplained pregnancy loss^3^. This dysfunction may involve imbalances in cytokines, growth factors and immunosuppressive factors at the maternal-fetal interface^4^. The role of cytokines in pregnancy loss is an emerging research field, which contributes to the understanding of many miscarriages without obvious etiologies.

Among the leukocytes that populate the maternal-fetal interface, CD4^+^ helper T cells (Th cells) can be activated and then differentiate into Th1, Th2, and Th17 cells and produce corresponding types of cytokines. It has been hypothesized that during pregnancy there is a subtle immunological shift to the Th2-type cytokine responses that would suppress potential harmful effects of the cell-mediated (Th1-type) immune system^5^. Thus, pregnancy has been labeled a ‘Th2 phenomenon’. Blois et al. showed that Th1 cytokines led to pregnancy loss in mice^6^, and Chaouat et al. demonstrated that this loss could be prevented by Th2 cytokines^7^. Inflammatory processes alter the balance of Th1 and Th2 cytokines and lead to a shift toward Th1 predominance. This abnormal shift of Th1/Th2 cytokines during early pregnancy initiates and intensifies the cascade of proinflammatory cytokine production involved in miscarriage. However, this concept cannot be applied to the whole steps of pregnancy, such as the implantation and preparation to parturition. In addition to the classical Th1 and Th2 cells, several novel effector T cell subsets have been recently identified, including Th17 cells. Wang et al. found an accumulation of Th17 cells in the peripheral blood of women with unexplained recurrent pregnancy loss (Recurrent Miscarriage) as compared to normal pregnant women^8^. These Th17 cells, which secrete interleukin-17A (IL-17A), IL-17F, IL-22, and IL-26, are thought to play a role in autoimmune diseases, allograft rejection, and inflammatory immune responses. It is now evident that Th17, Th1, and Th2 immunity, and regulatory T cell (Tregs) mediated immune regulation is essential for successful embryo implantation and establishment of pregnancy^9,10^.

The gut microbiota plays a crucial role in shaping and modulating the immune system and immune responses. Disruption of the commensal microbiota may alter the gut homeostatic balance and lead to gut microbial dysbiosis, which has been increasingly recognized as one of the risk factors in the development of inflammation, as well as autoimmune and immune-mediated diseases^11–13^. Gut microbial dysbiosis is linked to aberrant immune responses, in large part by producing small molecules that are often accompanied by abnormal production of inflammatory cytokines, such as IL-1β, IL-6, IL-17, IL-23, and interferon-γ (IFN-γ)^14,15^. The small molecules including their metabolites and components are not only necessary for immune homeostasis, but also influence the susceptibility of the host to many immune-mediated diseases and disorders. Short-chain fatty acids (SCFAs), the bacterially-derived molecules, have been reported to participate in the modulation of cytokines production and Tregs expansion^16,17^. Moreover, microbial metabolites can penetrate the epithelial barrier, allowing them to enter and accumulate in the host circulatory system where they are sensed by immune cells^18^. In pregnancy, many of the immunological and metabolic changes that occur at the placental interface serve to inhibit rejection of the fetus. Koren et al. have reported that the gut microbial community composition and structure are profoundly altered in the third trimester (T3), and the transfer of T3 microbiota induces inflammation in germ-free recipient mice to a great extent than that in first-trimester (T1) microbiota^19^. Recently, a nested case–control study indicated that vaginal bacterial composition in first-trimester miscarriage was associated with reduced *Lactobacillus spp*. abundance. Depletion of *Lactobacillus spp*. might lead to increased richness and diversity and colonization by potential pathogens^20^, and have been associated with the miscarriage^21,22^. However, the contribution of gut host-microbial interactions in promoting inflammatory cytokines production and other immune changes at early stages of pregnancy remains to be evaluated. Further, the underlying mechanisms of the correlation between gut microbial dysbiosis and the disturbance of cellular immune function in miscarriage patients remain inconclusive.

Given the effect of gut microbiota modulation on immune responses, we hypothesized that the imbalanced gut microbiota and their metabolites can be linked to the immune dysfunction in pregnancy loss. In the current study, we aimed to examine the network between gut microbial community composition, microbial metabolites, and proinflammatory cytokine responses by performing multi-omics analyses of subjects that had unexplained pregnancy loss versus controls who underwent elective abortion.

## Results

### Characteristics of study participants

The flow chart displays the enrollment and analysis process (Supplementary Fig. S1). Participants who indicated a reproductive tract infection and recent antibiotic treatment on the questionnaire were removed from the microbiota study. 41 miscarriage patients and 19 controls were included for 16S rRNA gene sequencing. The general characteristics of the study participants, including age, pre-pregnancy body mass index (BMI), education level, smoking status, alcohol-drinking status, reproductive tract infection, antibodies screen, and medical history are presented in Table 1. No substantial differences in these characteristics were found between the miscarriage cases and controls.

**Table 1:** Demographic and clinical characteristics of women with miscarriage and the controls.

Variables	Miscarriage (*n* = 41)	Control (*n* = 19)	*P* value
Age (years)^a^	31.3 ± 5.0	32.4 ± 4.7	0.43
Pre-pregnancy BMI (kg/m^2^)	21.5 ± 2.7	22.4 ± 4.2	0.39
Education			
Illiterate	0 (0%)^b^	0 (0%)	NE^c^
High school or lower	4 (10%)	4 (21%)	0.27
College	31 (76%)	14 (74%)	0.87
Postgraduate or higher	6 (14%)	1 (5%)	0.54
History of smoking	0 (0%)	0 (0%)	NE
History of drinking	0 (0%)	0 (0%)	NE
Bacterial vaginosis	0 (0%)	0 (0%)	NE
Ureaplasma urealyticum	0 (0%)	0 (0%)	NE
Chlamydia trachomatis	0 (0%)	0 (0%)	NE
Trichomoniasis	0 (0%)	0 (0%)	NE
Colpomycosis	0 (0%)	0 (0%)	NE
Virus			
HIV+	0 (0%)	0 (0%)	NE
HPV+	0 (0%)	0 (0%)	NE
Syphilis	0 (0%)	0 (0%)	NE
Antibody			
Anticardiolipin antibody (IgA, IgM, and IgG)	0 (0%)	0 (0%)	NE
Anti DNA antibody (single and double stranded)	0 (0%)	0 (0%)	NE
Anti ENA (Extractable nuclear antigen, 7 subtypes) antibody	0 (0%)	0 (0%)	NE
Irregular antibody	0 (0%)	0 (0%)	NE
Medical history			
Endometriosis	0 (0%)	0 (0%)	NE
Uterine fibroids	4 (8%)	1 (5%)	0.93
Endometrial polyps	0 (0%)	0 (0%)	NE
Intrauterine adhesion	0 (0%)	0 (0%)	NE
Ovarian cysts	0 (0%)	0 (0%)	NE
Pelvic inflammation	0 (0%)	0 (0%)	NE
DUB (Dysfunctional Uterine Bleeding)	0 (0%)	0 (0%)	NE
Rheumatoid arthritis	0 (0%)	0 (0%)	NE
SLE(Systemic Lupus Erythematosus)	0 (0%)	0 (0%)	NE

^a^Data are presented as mean ± SD.

^b^Data are presented as *n* (%).

^c^*NE* not estimable (due to nullity of category in both groups).

### Decreased bacterial diversity in fecal microbiota associated with miscarriage patients

Overall, the present 16S rRNA gene-targeted sequencing yielded between 14,982 and 36,470 valid tags with average lengths ranging from 423.18 to 433.71 bp. Following taxonomic assignment, 2835 operational taxonomic units (OTUs) were obtained, and these identified OTUs belong to 16 phyla, 30 classes, 55 orders, 96 families, and 268 genera. The species accumulation curve and the rarefaction curve (Supplementary Fig. S2) of all samples supported the adequacy of the sampling efforts. After false discovery rate (FDR) correction (FDR < 0.05), 859 OTUs, 7 phyla, 9 classes, 13 orders, 14 families, and 48 genera significantly differed between the cases and controls.

**Fig. 1:**
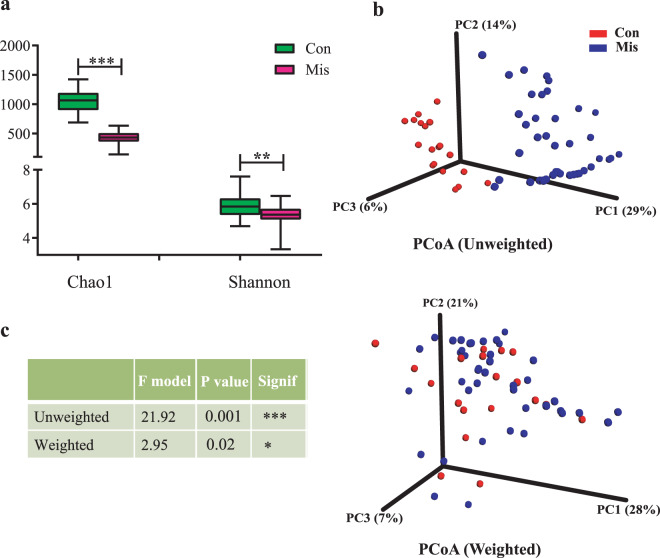
Gut microbiota diversity analyses. **a** Species diversity differences between the miscarriage (Mis) and control (Con) groups were estimated by the Chao1, and Shannon indices. The bar are shown as mean ± SD. **p < 0.01, ***p < 0.001. **b** PCoA plot base of the relative abundance of OTUs showing bacterial structural clustering. (i) Unweighted UniFrac PCoA plots; (ii) Weighted UniFrac PCoA plots. Miscarriage group (blue dots), Con group (red dots), where dots represent individual samples. **c** Adonis analysis of statistical summary of different groups. F Model, represents *F*-test value; *p < 0.05, ***p < 0.001.

To evaluate the differences in bacterial diversity between the two groups, sequences were aligned to estimate alpha diversity using Chao 1 and Shannon index and beta diversity using principal coordinate analysis (PCoA) plots based on the unweighted and weighted UniFrac distance. Chao 1 is an index of species richness, unrelated to abundance and evenness^23^. The Shannon index is related to not only species richness but also species evenness. Both Chao1 estimators and Shannon index were significantly decreased in the miscarriage group relative to the control group (*p* < 0.001 and *p* < 0.01, respectively), indicating a lower richness and evenness of gut bacteria in miscarriage patients (Fig. 1a). We then analyzed the beta diversity of the two groups. Both the unweighted and weighted PCoA plots revealed that the gut microbiota in subjects with miscarriage clustered significantly compared to that of the controls (Fig. 1b, c). These results indicate that the diversity of gut microbiota is significantly lower with different microbiota profile in the miscarriage patients than the controls.

### Alterations in the composition of fecal microbiota associated with miscarriage

**Fig. 2:**
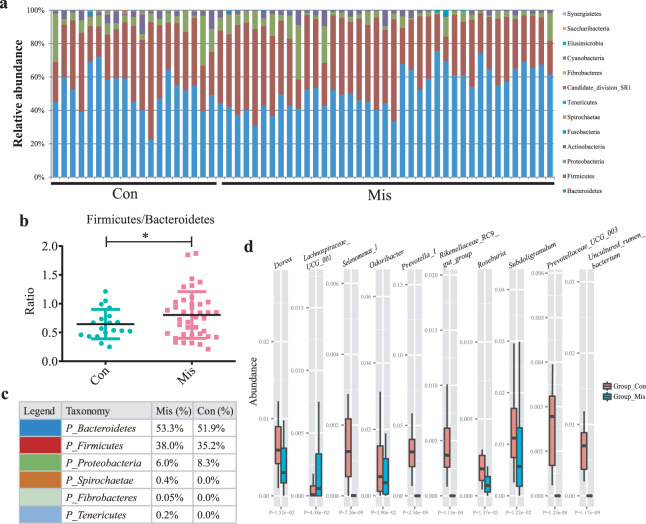
Gut microbial dysbiosis in miscarriage patients. **a** Relative abundance of the main bacterial phylum in each group. **b** The ratio of *Firmicutes*/*Bacteroidetes* in each group. Data are shown as mean ± SD. *p < 0.05. **c** Component proportion of bacterial phylum in each group. *n* = 41 for the miscarriage (Mis) group and *n* = 19 for the control (Con) group. **d** The top 10 significantly different genera in the relative abundances between the control and miscarriage groups; Kruskal–Wallis, all *p* < 0.05.

*Bacteroidetes* was the most predominant phylum, accounting for 53.3% and 51.9% of the OTUs in the miscarriage and control groups, respectively. *Firmicutes* was enriched in the miscarriage group compared to the control group, whereas *Proteobacteria* was enriched in the control group (Fig. 2a). Given that an upregulated *Firmicutes*/*Bacteroidetes* ratio has been suggested as an indicator of several pathological conditions^24^, the ratio was of 0.65 in the controls and 0.80 in the cases (*p* = 0.039), indicating that a pathological change occurred in miscarriage patients (Fig. 2b). In addition, at the phylum level, the abundances of *Spirochaetae* (*p* < 0.001), *Fibrobacteres* (*p* < 0.001), and *Tenericutes* (*p* < 0.001) were significantly increased in the miscarriage group than in the control group (Fig. 2c).

**Fig. 3:**
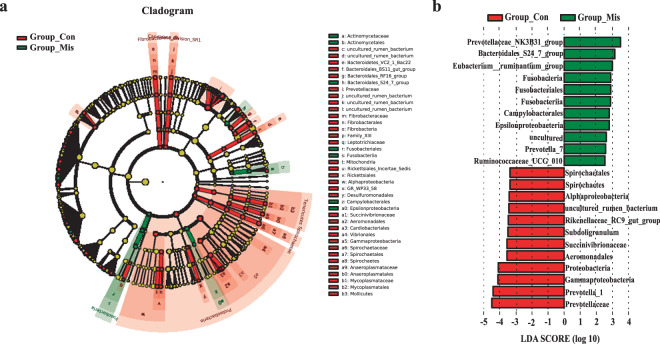
Compositions of different species in the control and miscarriage groups by LEfSe analyses. **a** Taxonomic representation of statistically and biologically consistent differences between the control and miscarriage groups. Significant differences are represented with different colors (red and green represent core microbes in the control (Con) and miscarriage (Mis) groups, respectively; yellow represents microbes shared between the control and miscarriage groups). **b** Histogram of LDA scores for differentially abundant genera between the control and miscarriage groups.

At the genus level, 48 genera of bacteria, including *Prevotella_1*, *Prevotellaceae_UCG_003*, *Roseburia*, and *Selenomonas_1*, were significantly reduced in abundance in the miscarriage group (Fig. 2d, and Supplementary Table S1). Considering that this discriminant analysis did not distinguish the predominant taxon, linear discriminant analysis coupled with effect size measurements (LEfSe) was used to generate a cladogram to identify the specific bacteria associated with miscarriage (Fig. 3a). It was shown that several opportunistic pathogens including *Prevotellaceae_NK3B31_group*, *Bacteroidales_S24_7_group*, and *Eubacterium ruminantium_group* were all significantly overrepresented (all LDA scores (log10) >3.0) in the feces of miscarriage patients, whereas *Prevotellaceae*, *Prevotella_1*, and *Gammaproteobacteria* were the most abundant microbiota in the control group (LDA scores (log10) >4.0) (Fig. 3b). These results indicate that the alterations in the composition of fecal microbiota were associated with miscarriage.

### Fecal metabolites profiles were altered in miscarriage patients

The fecal metabolome is a functional readout of the gut microbiome. Fecal metabolic profiling is a novel tool for exploring links between microbiota composition and host phenotypes^25^. LC/MS analysis was used to obtain the fecal metabolic profiles of the 20 subjects. To avoid potential selection bias, we performed a random selection. To validate the representativeness of these selected samples, we compared the demographic and clinical characteristics, the diversity and microbiota composition in the selected 10 cases and that in all cases (10 selected cases vs all 41 cases) or 10 controls samples with that in all control samples (10 selected controls vs all 19 controls). No significant differences were found (Supplementary Tables S2 and S3). To further validate the representative of these selected samples, we compared the diversity and fecal microbiota profiles in the selected 10 case samples with that in the 10 selected control samples. The significant differences between these two selected groups resemble the comparison between all cases and controls (Supplementary Fig. S3).

**Fig. 4:**
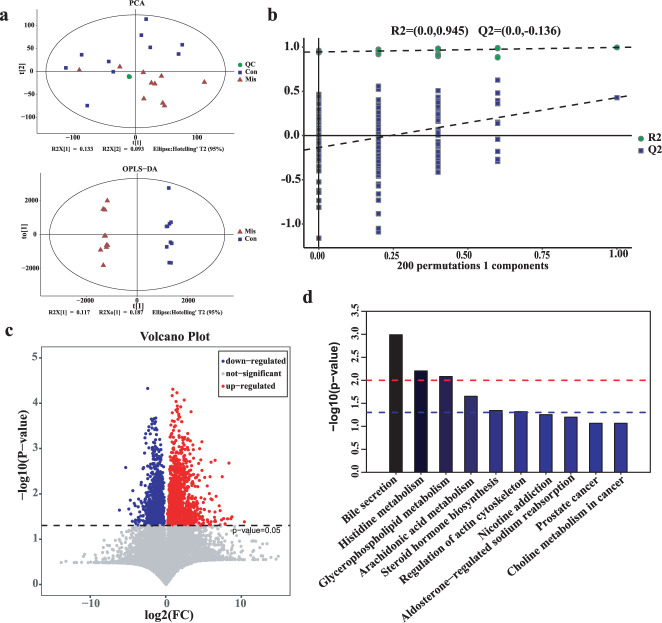
Fecal metabolomics for quantification of metabolites in the miscarriage and control groups. **a** PCA and OPLS-DA score plots for differentiating the metabolites in each group. **b** The corresponding permutation test (200 times) for the OPLS-DA model. **c** Volcano plot showing the differentially accumulated metabolites in the miscarriage (Mis) and control (Con) groups. Red dots indicate the metabolites upregulated in the miscarriage group, and blue dots indicate the metabolites down-regulated in the miscarriage group. **d** Enriched KEGG pathways in the miscarriage group compared with the control group.

The quality control (QC) samples in the principle component analysis (PCA) score plot overlapped, which indicates that samples behaved stably for the duration of the run. Using PCA and (orthogonal) partial least-squares-discriminant analysis (OPLS-DA), we found that the miscarriage group was completely separated from the control group (R2Y (cum) = 0.996, Q2 (cum) = 0.428), demonstrating that metabolic disturbances exist in these two groups. Significant differences of fecal metabolites were observed in the miscarriage and control groups (Fig. 4a). The permutation test indicated that the analytical platform exhibited excellent stability and repeatability (R2 = 0.945, Q2 = -0.136), and can be utilized in subsequent metabolomics research (Fig. 4b). In total, 23706 metabolites were detected in these samples. Based on the differential screening strategy, 239 discriminating metabolites were found in the miscarriage group compared with the control group (Fig. 4c and Supplementary Table S4). The Kyoto Encyclopedia of Genes and Genomes (KEGG) pathway enrichment analyses indicated that these differentially presented metabolites were related to (1) bile secretion (5alpha-androstane-3alpha-ol-17-one sulfate, Deoxycholic acid 3-glucuronide, TXB2, L-Carnitine, and acetylcholine); (2) histidine metabolism (1,4-Methylimidazoleacetic acid, ergothioneine, and imidazolepropionic acid); (3) glycerophospholipid metabolism (acetylcholine, lysoPC(22:1(13Z)), and sn-3-O-(geranylgeranyl)glycer); (4) arachidonic acid metabolism pathways (TXB2, 15-Deoxy-d-12,14-PGJ2, and 12(S)-HETE), and (5) steroid hormone biosynthesis (5alpha-androstane-3alpha-ol-17-one sulfate, cortisone, and 7a-Hydroxydehydroepiandrosterone) (Fig. 4d, *p* < 0.05).

### Clustering and multivariate analyses reveal distinct metabolites in the miscarriage and control groups

**Fig. 5:**
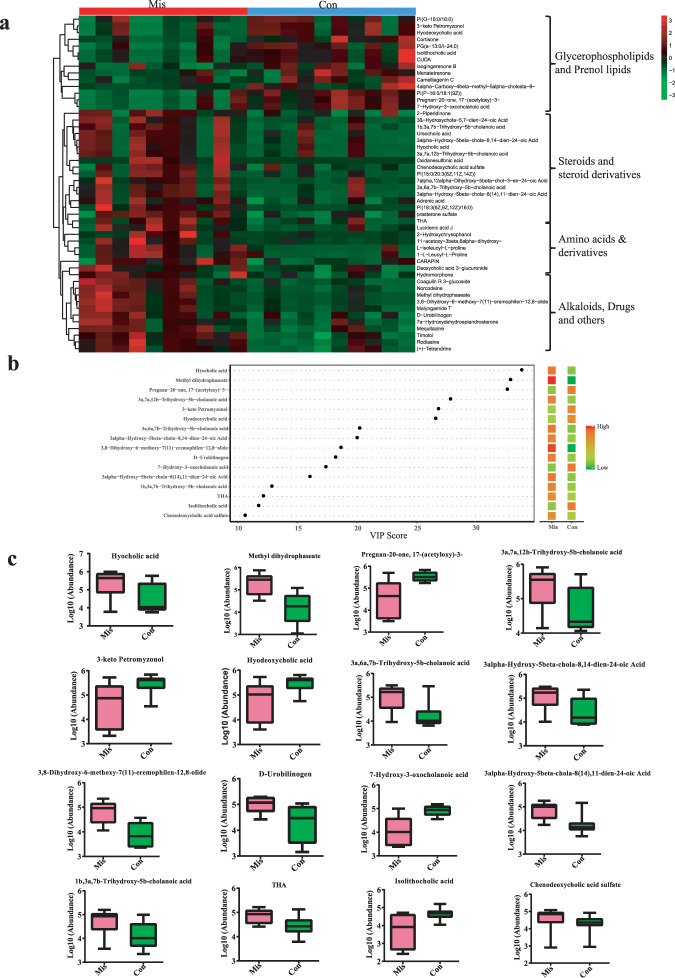
Distinct metabolites, as identified by clustering and multivariate analyses, between the miscarriage (Mis) and control (Con) groups. **a** Hierarchical clustering analysis (HCA) for the miscarriage and control group metabolites based on their z-normalized abundances. **b** The discriminatory metabolites of top 16 VIP scores which obtained from the OPLS-DA models. **c** Abundance comparisons of metabolites between the miscarriage and control groups. The bar are shown as mean ± SD.

The hierarchical clustering analysis (HCA) of the metabolites that differed between the miscarriage and control groups revealed four large clusters: (i) glycerophosphplipids and prenol lipids, which showed higher abundances in the control group than in the miscarriage group, (ii) steroids and steroid derivatives, (iii) amino acids and derivatives, and (iv) alkaloids, drugs and other metabolites, which showed higher abundances in the miscarriage group than in the control group (Fig. 5a). The variable importance in the projection (VIP) values, which were obtained by OPLS-DA analysis, indicate the importance of metabolites for interpreting the differences. The presence of several metabolites, such as hyocholic acid, methyl dihydrophaseate, pregnan-20-one,17-(acetyloxy)-3-, 3a, 7a, 12b-Trihydroxy-5b-cholanoic acid, 3-keto petromyzonol, and hydeoxycholic acid et al. were able to differentiate the cases from the controls (Fig. 5b). Specifically, the abundances of hyocholic acid, methyl dihydrophaseate, 3a, 7a, 12b-Trihydroxy-5b-cholanoic acid, 3a, 6a, 7b-Trihydroxy-5b-cholanoic acid, 3alpha-Hydroxy-5beta-chola-8,14-dien-24-oic acid, 3,8-Dihydroxy-6-methoxy-7(11)-eremophilen-12,8-olide, D-Urobilinogen, 1b,3a,7b-Trihydroxy-5b-cholanoic acid, THA, and chenodeoxycholic acid sulfate were significantly higher in the miscarriage patients than in controls (Fig. 5c). Taken together, our data clearly demonstrated that miscarriage patients had a unique fecal metabolome, suggesting that certain gut microbiota profiles and metabolites may be associated with miscarriage.

### Correlation analysis of fecal microbiota, proinflammatory cytokines, and metabolites

**Fig. 6:**
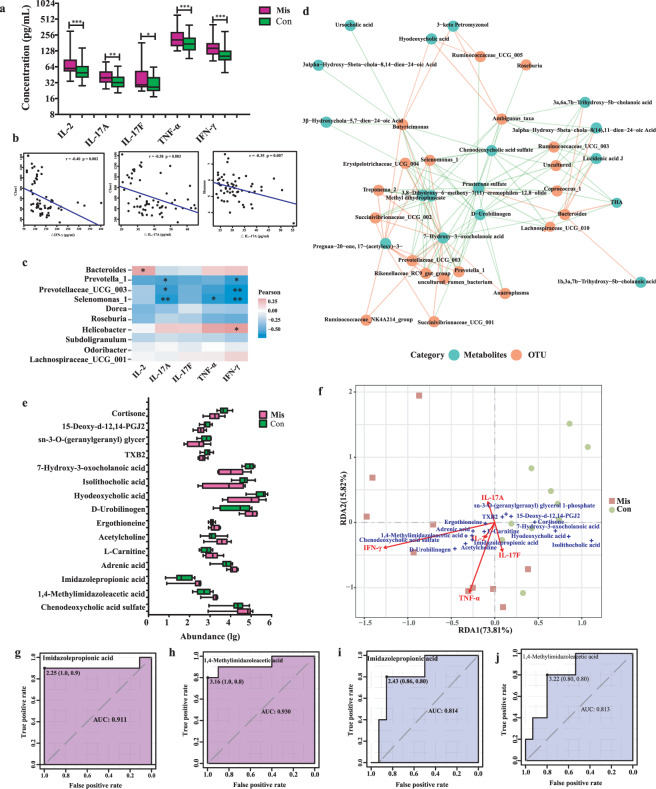
Correlation analyses of fecal microbiota and metabolites and the inflammatory cytokine profiles. **a** The inflammatory cytokine profiles in miscarriage patients (Mis, *N* = 41) and controls (Con, *N* = 19). The bar are shown as mean ± SD. *p < 0.05, **p < 0.01, ***p < 0.001. **b** Correlation between Chao1 index, Shannon index, and proinflammatory cytokines using Pearson’s linear correlation model. **c** Heatmap analysis of the correlation between the top 10 abnormal genera and proinflammatory cytokines. Orange represents positive correlations and blue negative correlations. *p < 0.05, **p < 0.01. **d** Network analysis of the correlation between abnormal genera and discriminatory metabolites. Red line represents positive correlations and green negative correlations. **e** Abundance profiles analysis of the microbe-associat**e**d metabolites. Data are shown as mean ± SD. Abundance (lg) means the abundance with log10 transformation. **f** RDA analysis of the correlation between inflammatory cytokines and discriminatory metabolites. **g-j** ROC analyses for metabolite abundance showing the high AUCs for imidazolepropionic acid and 1, 4-Methylimidazoleacetic acid in miscarriage patients (**g** and **h**) and their recurrent miscarriage (**i** and **j**).

Multiplex analysis revealed markedly increased serum levels of IL-2, IL-17A, IL-17F, tumor necrosis factor-α (TNF-α), and IFN-γ in miscarriage patients than those in the controls (Fig. 6a). Pearson analysis indicates that the Chao1 index was negatively associated with the changes in IL-17A, and IFN-γ, and the Shannon index was negatively associated with the changes in IL-17A (Fig. 6b). Moreover, the *Bacteroides* abundances were positively associated with the changes in IL-2; the *Helicobacter* abundances were positively associated with the changes in IFN-γ; the *Prevotella_1* and *Prevotellaceae_UCG_003* abundances were negatively associated with the changes in IL-17A and IFN-γ, and *Selenomonas_1* was negatively associated with the changes in IL-17A, TNF-α, and IFN-γ (Fig. 6c). As shown in the association network (Fig. 6d, and Table 2), the *Bacteroides* abundances were positively associated with the changes in THA, and lucidenic acid J; both the *Prevotella_1* and *Prevotellaceae_UCG_003* abundances were positively associated with the changes in 7-Hydroxy-3-oxocholanoic acid, and negatively correlated with those of 1,4-Methylimidazoleacetic acid, and imidazolepropionic acid; the *Selenomonas_1* was positively associated with the changes in 7-Hydroxy-3-oxocholanoic acid, and negatively associated with the changes in 1,4-Methylimidazoleacetic acid, imidazolepropionic acid, adrenic acid, and chenodeoxycholic acid sulfate.

**Table 2:** Correlation analyses between the genera and abnormal metabolites.

Metabolites	*Bacteroides*		*Prevotella_1*		*Prevotellaceae_UCG_003*		*Selenomonas_1*	
	*r*	*p*	*r*	*p*	*r*	*p*	*r*	*p*
THA	0.74	<0.001	−0.21	0.27	−0.27	0.26	−0.24	0.31
Lucidenic acid J	0.78	<0.001	−0.35	0.13	−0.38	0.10	−0.32	0.17
Ergothioneine	0.45	0.04	−0.37	0.10	−0.35	0.13	−0.31	0.18
7-Hydroxy-3-oxocholanoic acid	−0.48	0.03	0.61	0.004	0.69	<0.001	0.66	0.001
Cortisone	−0.27	0.26	0.46	0.04	0.48	0.03	0.46	0.04
1,4-Methylimidazoleacetic acid	0.31	0.11	−0.65	0.002	−0.65	0.002	−0.60	0.005
Imidazolepropionic acid	0.34	0.09	−0.68	0.001	−0.67	0.002	−0.66	0.002
Acetylcholine	0.26	0.26	−0.45	0.004	−0.38	0.09	−0.48	0.03
12-Oxo-20-carboxy-leukotriene B4	0.49	0.03	−0.61	0.004	−0.62	0.003	−0.60	0.006
Adrenic acid	0.50	0.02	−0.43	0.06	−0.48	0.03	−0.49	0.03
16,16-dimethyl-6-keto Prostaglandin E1	−0.35	0.13	0.54	0.01	0.59	0.007	0.62	0.003
Chenodeoxycholic acid sulfate	0.41	0.08	−0.42	0.06	−0.42	0.06	−0.48	0.03

*r* represents the correlation coefficient, *p* represents the *p* values.

In addition, we identified some microbial-associated metabolites which were significantly enriched in the bile secretion, histidine metabolism, and arachidonic acid metabolism pathways in miscarriage group, suggesting a correlation with an imbalance in gut microbiota or increased serum cytokines. The correlation analysis between these metabolites and cytokines demonstrated that hyodeoxycholic acid, isolithocholic acid, 7-Hydroxy-3-oxocholanoic acid, TXB2, sn-3-O-(geranylgeranyl) glycer, 15-Deoxy-d-12,14-PGJ2, and cortisone, which were decreased in miscarriage patients, were negatively associated with the changes in serum levels of IL-17A, IL-17F, TNF-α, and IFN-γ, while the increased fecal chenodeoxycholic acid sulfate, 1,4-Methylimidazoleacetic acid, imidazolepropionic acid, adrenic acid, L-Carnitine, acetylcholine, ergothioneine, and D-Urobilinogen in the miscarriage patients were positively associated with the changes in IL-17A, IL-17F, TNF-α, and IFN-γ (Fig. 6e, f, and Table 3). A receiver operating characteristic (ROC) curve analysis indicated that imidazolepropionic acid (area under the curve (AUC), 0.911; Fig. 6g) and 1, 4-Methylimidazoleacetic acid (AUC, 0.930; Fig. 6h) were significantly associated with miscarriage.

**Table 3:** Correlation analyses between the abnormal metabolites and inflammatory cytokines.

Metabolites	Correlation coefficient (r)				
	IL-2	IL-17A	IL-17F	TNF-α	IFN-γ
Hyocholic acid	0.27	0.36	0.29	0.26	0.39
Ursocholic acid	0.29	0.35	0.36	0.28	0.35
Chenodeoxycholic acid sulfate	0.20	0.32	0.45*	0.32	0.35
Hyodeoxycholic acid	−0.09	−0.20	−0.05	−0.13	−0.65**
Isolithocholic acid	−0.09	−0.04	−0.11	−0.23	−0.50*
1,4-Methylimidazoleacetic acid	0.13	0.45*	0.41	0.58**	0.59**
Imidazolepropionic acid	0.15	0.47*	0.47*	0.59**	0.53*
Adrenic acid	0.04	0.43	0.42	0.36	0.38
7-Hydroxy-3-oxocholanoic acid	−0.30	−0.62**	−0.35	−0.44	−0.46*
Lucidenic acid J	0.17	0.23	0.15	0.14	0.44
THA	0.21	0.27	0.17	0.16	0.43
Deoxycholic acid 3-glucuronide	0.20	0.06	0.01	0.20	0.40
L-Carnitine	0.17	0.30	−0.06	0.34	0.54*
Acetylcholine	0.07	0.11	0.07	0.35	0.52*
5alpha-androstane-3alpha-ol-17-one sulfate	0.18	0.23	0.28	0.10	0.31
TXB2	−0.12	-0.47*	−0.32	−0.49*	−0.40
Ergothioneine	0.11	−0.02	−0.01	0.08	0.50*
LysoPC(22:1(13Z))	0.20	0.15	0.34	0.28	0.34
sn-3-O-(geranylgeranyl) glycerol 1-phosphate	−0.30	−0.54*	−0.33	−0.39	−0.18
15-Deoxy-d-12,14-PGJ2	−0.23	−0.50*	−0.26	−0.55*	−0.52*
12(S)-HETE	−0.17	−0.02	−0.37	−0.33	−0.20
Cortisone	−0.19	−0.26	−0.22	−0.34	−0.54*
D-Urobilinogen	0.12	0.25	0.16	0.31	0.46*
12-Oxo-20-carboxy-leukotriene B4	0.19	0.39	0.39	0.42	0.26
7a-Hydroxydehydroepiandrosterone	0.16	0.14	0.09	0.26	0.37
16,16-dimethyl-6-keto Prostaglandin E1	−0.28	−0.37	−0.23	−0.42	−0.32

The asterisk represents the statistical significance between metabolites and cytokines. *p < 0.05, **p < 0.01.

*IL* interleukin, *TNF-α* tumor necrosis factor-α, *IFN-γ* interferon-γ.

To investigate whether patients with certain characteristics of gut microbiota and their metabolites are more susceptible to repeated pregnancy loss or infertility, we conducted a follow up survey. Our follow-up results demonstrated that 17 participants had a recurrent miscarriage or unsuccessful pregnancy (Supplementary File 1). The ROC analysis also showed higher AUCs for imidazolepropionic acid (0.814; Fig. 6i) and 1, 4-Methylimidazoleacetic acid (0.813; Fig. 6j) for the recurrent miscarriage. Thus, these results reveal a link between the distinct metabolites (e.g., imidazolepropionic acid and 1, 4-Methylimidazoleacetic acid) and Th17 immunity in the miscarriage patients.

## Discussion

To fill the knowledge gap of the interaction role of host and microbiota in unexplained miscarriages and provide initial insights into this aspect, we investigated the network between gut microbiota, fecal metabolites, blood cytokines, and unexplained miscarriages. We found that the diversity and composition of gut microbiota and metabolites profiles were significantly altered in miscarriage patients. Further analyses revealed that these alterations in gut microbiota were related to the increased Th1- and Th17-related cytokines. The metabolites profiles revealed potential links between gut microbiota and the changes in cytokines, and were associated with recurrent miscarriage or subfecundity in the cohort of miscarriage patients. To the best of our knowledge, this is the first study to investigate the association of gut microbiota, fecal metabolites profiles and proinflammatory cytokines in miscarriage patients.

The Th1 response, especially IL-2, TNF-α, and IFN-γ, is harmful to the survival of the conceptus^26,27^. A significantly higher level of the Th1 cytokine IFN-γ was present in women with recurrent miscarriage compared with to these with normal pregnancies^28^. TNF-α has been reported to inhibit trophoblast invasion, and increased levels of TNF-α have been reported in women with miscarriage^29,30^. Moreover, Liu et al. found that Th17 cells were significantly increased in women with recurrent miscarriage compared with women with normal healthy pregnancies^31^. In mice, abnormal elevation of IL-17 at the maternal-fetal interface led to a miscarriage, while administration of an anti-IL-17 antibody prevented unexplained recurrent miscarriage^32^. The current study also found that serum levels of IL-2, IL-17A, IL-17F, TNF-α, and IFN-γ were markedly increased in miscarriage patients compared to the controls. These results suggest that women with miscarriage have a propensity for proinflammation via Th1- and Th17-mediated immunity.

The gut microbiota of a host is a crucial factor for shaping and modulating the immune responses^33–35^. Gut microbial dysbiosis is a risk factor in the development of inflammation^36,37^. Reduction of gut microbial diversity has been linked to an increased risk of gastrointestinal diseases and proinflammatory characteristics^38,39^, and low gut bacterial richness is a common hallmark of chronic disease^40^. It was also reported that reduced vaginal *Lactobacillus spp*. abundance drives activation of inflammatory pathways that negatively modify endometrial receptivity and implantation^20,41,42^. These effects are not confined to local responses, but rather, integrated with maternal systemic immunity^43^. The underlying connections between gut microbiota, the reproductive tract microbiota, and miscarriage need to be further clarified. During pregnancy, the gut microbial within-subject (α) diversity is similar to that of the non-pregnant state at T1, but reduced at T3, after comparing to the 16S rRNA gene sequencing data from the Human Microbiome Project (HMP)^19^. This shift from T1 to T3 includes an increase in the levels of the proinflammatory cytokines IL-2, IL-6, IFN-γ, and TNF-α^19,44^. Here we report a negative association between systemic Th1- and Th17-associated cytokines and reduced gut microbial diversity during T1, indicating a correlation between gut microbial diversity and the increased proinflammatory cytokines in this cohort of miscarriage patients. Previous study reported that the increased ratio of *Firmicutes* to *Bacteroidetes* is related to chronic inflammation^45^. In our study, we observed both the reduced gut microbiota and the increased ratio of *Firmicutes* to *Bacteroidetes* in the miscarriage group during T1, indicating that the proinflammatory effects of the microbiome in patients with miscarriage are likely caused by holistic dysbiosis, rather than by a specific pathogen.

In addition to the decreased diversity, we also found that *Prevotella_1*, *Prevotellaceae_UCG_003*, and *Selenomonas_1*, genera known as the dominant bacteria community in the gastroenteric environment of healthy humans, were significantly reduced in miscarriage patients. Similarly, decreased abundances of *Prevotellaceae* were also found in feces of individuals with chronic kidney disease^46^, colorectal cancer^47^, and Parkinson’s disease^48^. Studies have reported that the death of *Prevotella* can result in systemic inflammation by inducing increased plasma lipopolysaccharides (LPS) levels^49,50^. Moreover, a decrease in *Prevotella* and *Prevotellaceae* may cause a degeneration in the abundance of SCFAs, particularly butyrate, which can serve as the energy substrates for epithelial cells of the gut^51^. Thus, a decrease in *Prevotella* and *Prevotellaceae* leads to the reduction of inducible regulatory T cells (iTregs) and activation of proinflammatory cells^52,53^. The negative correlation between the decreased abundance of *Prevotella_1*, *Prevotellaceae_UCG_003* and serum levels of IL-17A found in our study provide further evidence for this association. Notably, *Prevotellaceae_NK3B31_group* was significantly overrepresented in the feces of miscarriage patients. The genus *Prevotellaceae-NK3B31 group* was reported to be positively associated with the cortisol level^54^, which was related to higher proinflammatory cytokines^55^. Many previous studies have reported on the benefits of *Bifidobacterium* for the reduction of inflammation. Miyauchi et al. found that *Bifidobacterium longum subsp*. alleviated intestinal inflammatory reactions through inhibition of IL-17A production by intestinal epithelial cells^56^. Although no significant decrease of *Bifidobacterium* abundance was found in miscarriage patients in our study, a positive correlation of *Prevotella_1* and *Bifidobacterium* (Supplementary Fig. S4) might indicate a regulatory role for *Prevotella_1* on Th17 cytokines. Moreover, we found that both *Prevotella_1* and *Prevotellaceae_UCG_003* correlated highly with enriched imidazolepropionic acid and 1, 4-Methylimidazoleacetic acid, both of which had higher abundance in the miscarriage group. And these two metabolites were positively associated with the level of IL-17A and IL-17F. These data suggest that the gut microbiome has biologically relevant effects on the modulation of Th17 cytokine production in women with early miscarriage.

Modulation of host defense by the microbiota may be exerted mainly through the release of intermediary common mediators (such as metabolites) rather than direct interaction between specific microorganisms and immune cells. An important role for metabolites in microbiota-cytokine interactions is supported by the fact that a large proportion of the metabolites in the blood originate from the gut^57,58^, and our findings of the strong impact of microbial metabolic processes on cytokines production. We observed that several microbiota-associated metabolites that were enriched in the bile secretion pathway, were also significantly altered in the feces of miscarriage patients. It is well-documented that bile acid plays a potent regulation role on intestinal immunity cells^59^. A recently published paper in *Nature* demonstrated that bile acid metabolites can control Th17 and Treg cell differentiation^60^. In the present study, we found negative associations between hyodeoxycholic acid, isolithocholic acid, and TXB2, which were significantly decreased in the miscarriage group, with serum cytokines, IL-17, TNF-α, and IFN-γ, and positive associations for chenodeoxycholic acid sulfate, L-Carnitine, acetylcholine, and cholic acid metabolites. Our results suggest that the bile acid metabolism of the gut microbiota strongly influences Th17-associated cytokine production. Moreover, we found that the fecal concentrations of 1, 4-Methylimidazoleacetic acid, and imidazolepropionic acid that were enriched in histidine metabolism were also significantly increased in the miscarriage group and aligned well with the increased IL-17, TNF-α, and IFN-γ in this group. Microbially produced imidazole propionate, which has been shown to have systemic effects and is present at higher concentrations in subjects with type 2 diabetes, impairs insulin signaling through mammalian target of rapamycin1 (mTORC1)^61^. A high level of imidazole propionate was reported to be involved in immune activation and low-grade inflammation^62,63^. Intriguingly, the ROC analysis indicated that the two imidazole propionic acids we examined were associated with miscarriage and had a positive predictive value for recurrent miscarriage. Collectively, these results indicate that the gut microbiota has biologically relevant effects on the modulation of Th17 cytokine production through their metabolites in miscarriage.

The strength of this study is that we applied 16S rRNA gene sequencing, metabolomics, and host serum cytokines networks, which allowed us to gain more information about host–gut microbiota metabolic interactions in response to the imbalanced cytokines network in unexplained miscarriage. One limitation of this study is that we did not obtain completed questionnaires about the dietary habits of the participants, and therefore could not determine if the diet was a factor in the gut bacterial dysbiosis. In addition, we cannot rule out other etiologies of miscarriage patients such as PCOS and insulin resistance, which might be indirectly associated with the proinflammatory cytokines. Further, the database for identification of metabolites in this study does not include the compound spiking and MS2, which may lead to the risk of identification bias. It is also important to note that the sample size is relatively small and the identified networks are associative. Thus, further large cohort studies are required to establish a causal relation between gut microbiota, fecal metabolites, and blood levels of cytokines and miscarriage. Despite some limitations, this study opens new avenues towards understanding the gut microbiome-metabolome-immunity-miscarriage connections.

In conclusion, we demonstrated an association between gut bacterial dysbiosis and a Th1/Th17-mediated proinflammatory state in miscarriage patients with unknown etiology. This study provided insights into the potential roles of gut microbiota in the pathogenesis of some unexplained miscarriages and the potential underlying mechanisms. Further studies are warranted to determine the casual relationship between gut microbiota and inflammation-induced miscarriage and whether the microbiota-associated metabolites are the underlying mechanism of the cytokine network imbalance through larger prospective cohort studies and animal studies.

## Methods

### Participant enrollment

Eligible cases were women (i) who were less than 35 years of age with a pre-pregnancy BMI of 18.5–23.9 kg/m^2^ (normal reference value standards of BMI for Chinese); (ii) who had no successful pregnancies (their miscarriages occurred before 14 weeks of gestation); and (iii) whose current male partner had normal semen testing (sperm density > 15 million/mL, and sperm motility > 60%) by computer-assisted analysis. Age and gestational week-matched controls were women with normal early pregnancy who elected to have an abortion. All controls had at least one successful pregnancy and no history of miscarriage, preterm labor, or pre-eclampsia. Subjects with high-risk factors of miscarriage were excluded. Exclusion criteria were: (i) abnormality of the uterus or cervical incompetence confirmed by transvaginal ultrasonography. (ii) karyotype abnormality in either the parents or the embryo. Karyotyping abnormity was defined as abnormal chromosome number (such as trisomy, polyploidy, and monosomy X) and abnormal chromosome structure (either a balanced reciprocal translocation or a Robertsonian translocation). (iii) luteal phase defect (diagnosed by basal body temperature in combination with serum progesterone levels < 10 ng/mL), hyperprolactinemia, or hyperandrogenemia. (iv) presence of autoantibodies-like antinuclear antibodies (ANA), anticardiolipin antibodies (ACL), irregular antibody, and extractable nuclear antigens antibodies associated with systemic lupus erythematosus (SLE). In total, 41 cases and 19 controls were recruited and samples were collected between September 2017 and October 2018. Follow-up of pregnancy outcomes was conducted for all participants at the end of 2019. The study protocol was approved by the Ethics Committees of Xinhua Hospital affiliated to Shanghai Jiao Tong University School of Medicine (XHEC-C-2017-073). Informed consents were obtained from all participants.

### Stool sampling, and DNA extraction

Women in the first trimester of pregnancy with an unexplained miscarriage or that underwent elective abortion were recruited. A detailed questionnaire including details on demographic information, medical history, gynecological and obstetric history was completed. Participants were instructed to collect stool samples at home using sterilized containers and store their samples in a refrigerator right after collection and deliver the samples in an ice bag to the hospital. Sampling for these participants who failed to collect samples at home were performed in the hospital before curettage. Participants who have taken antibiotics, probiotics, prebiotics, or synbiotics two months prior to sample collection were excluded. A metadata including detailed information on the sample collection and follow-ups for each participant was summarized in Supplementary File 1. In total, 41 cases and 19 controls were included in 16S rRNA gene sequencing. Genomic DNA was extracted from stool samples using the QIAamp DNA Stool Mini Kit (QIAGEN, Dusseldorf, Germany) according to the manufacturer’s instructions. The quality of genome DNA was evaluated with 1% agarose gel, and DNA concentrations were determined using NanoDrop spectrophotometry (NanoDrop, Germany).

### High-throughput 16S rRNA gene sequencing

The V3-V4 variable regions of the 16S rRNA gene were amplified using bacterium-specific primers 343F (5’-TACGGRAGGCAGCAG-3’) and 798R (5’-AGGGTATCTAATCCT-3’)^64^. Amplicon quality was visualized using gel electrophoresis, purified with AMPure XP beads (Agencourt AMPure XP, Beckman), and then amplified for another round of PCR. After purified with the AMPure XP beads again, the final amplicon was quantified using Qubit dsDNA assay kit (Life Technologies, USA). Equal amounts of purified amplicon were pooled and sequenced on an Illumina MiSeq PE300 with two paired-end read cycles of 300 bases each. (Illumina Inc., San Diego, CA; OEbiotech Co., Ltd., Shanghai, China). Briefly, to sequence the 16S V3-V4 region, primers were designed against the surrounding conserved regions. These primers were tailed with sequences to incorporate Illumina adapters with indexing barcodes. The sample containing pooled barcoded samples was loaded onto the MiSeq reagent cartridge, and then onto the instrument along with the flow cell. Automated cluster generation and paired-end sequencing with a 13-cycle index read was carried out without any further user intervention.

### Bioinformatic analysis of the fecal microbiota

The raw data obtained from high-throughput sequencing were stored in FASTQ format. Low-quality sequences that had an average quality score below 20 were cut off using Trimmomatic software^65^. After trimming, paired-end reads were assembled with FLASH software^66^. The parameters of assembly were as follows: 10 bp of minimal overlapping, 200 bp of maximum overlapping and a 20% maximum mismatch rate. Further de-noising including the removing of ambiguous base (N), sequences below 200 bp, and chimeras was performed with QIIME software (version 1.8.0)^67^. The obtained clean reads were then subjected to primer-sequence removal and clustering to generate OTUs with a 97% similarity cutoff using Vsearch software^68^. The most abundant one was selected as the representative read of each OTU. All representative reads were annotated and blasted against the Silva database (v. 123) using RDP classifier (at a confidence threshold of 70%). Alpha diversity (α-diversity) indices (including Chao1 estimators and Shannon index) evaluating gut microbial community richness and evenness were performed by Mothur^69^. PCoA based on the unweighted and weighted UniFrac distance was performed to compare the global microbiota composition (β-diversity) between the miscarriage and control groups, and statistically significant differences between the two groups were calculated by Adonis analyses^70^. LEfSe was applied to identify the bacterial species that differed between samples^71^.

### Fecal metabolic profiling and data analysis

Ten miscarriage patients and 10 matched control subjects were randomly chosen for the metabolomics study. The samples were processed in a randomized manner. Accurately weighed 60 mg fecal sample was transferred to a 1.5-mL Eppendorf tube, and then 20 μL each of L-2-chlorophenylalanine solution (0.3 mg/mL) and Lyso PC17:0 (0.01 mg/mL) were added as an internal standard. The QC samples (*n* = 4) were pooled ones in which aliquots of each sample (*n* = 20) were mixed together. Following ultrasonication and centrifugation, the supernatants from each tube were collected using crystal syringes, filtered through 0.22 μm microfilters, transferred into a liquid chromatography/mass spectrometry (LC/MS) glass vial, and analyzed by an ACQUITY UHPLC system (Waters Corporation, Milford, USA) coupled with an AB SCIEX Triple TOF 6600 System (AB SCIEX, Framingham, MA) as described previously^72^.

The acquired raw data were analyzed by the progenesis QI software (Waters Corporation, Milford, USA) using the following parameters: precusor tolerance at 5 ppm, fragment tolerance at 10 ppm, and product ion threshold at 5%. Metabolites were identified based on public databases such as the Human Metabolome Database (HMDB), Lipidmaps (v2.3), and METLIN. PCA and OPLS-DA models were carried out to visualize the metabolic alterations among experimental groups. A permutation test (*n* = 200) was performed to validate the model and avoid over fitting. HCA was applied on Pearson distances using PermutMatrix^73^. Differential metabolites contributing to the separation were identified using VIP value, fold change values and the corresponding *p* values. In general, metabolites with VIP > 1 were considered as relevant for interpreting the discrimination, a fold change value ≥1.5 or ≤ 0.667 was the cutoff for up or down-regulation in concentration, and *p* value < 0.05 was considered to be a significant difference.

### Cytokines quantification by flow cytometry

A bead-based multiplex panel assay (Biolegend, San Diego, CA, USA) was used for simultaneous quantification of 13 human cytokines including IL-2, -4, -5, -6, -9, -10, -13, -17A, -17F, -21, -22, IFN-γ, and TNF-α in the corresponding 41 vs 19 subjects. Briefly, the peripheral blood was collected and centrifuged for 20 min at 1000 × *g* to collect serum. Serum samples were diluted two folds with Assay Buffer before being tested. Next, 25 μL of mixed beads, detection antibodies, and streptavidin-phycoerythrin were added stepwise to each well. The plate was placed on a plate shaker for shaking at 500 rpm for 30 min at room temperature. After washing the beads three times with 200 μL of 1×Wash Buffer, the beads were resuspended in 150 μL of 1×Wash Buffer, placed on a plate shaker for 1 min and read on a flow cytometer (Supplementary Fig. S5).

### Statistical analysis

Data are presented as the mean ± standard deviation (SD). Statistical comparisons of continuous variables describing clinical characteristics were calculated using Student’s *t* test where data were normally distributed. Chi-square test was applied for categorical variables. For the bacterial abundance analysis, data were firstly normalized and one-way ANOVA or Kruskal–Wallis was used to test for the significant difference in genus relative abundance. Student’s *t* test was performed to analyze the differential in metabolites abundance between the miscarriage and control groups. Spearman’s rank test or Pearson’s correlation test was used to analyze the correlation between changes in genus relative abundance, fecal metabolites, and the levels of serum cytokines. *P* < 0.05 was considered statistically significant, and the *p* value was adjusted by the Benjamini–Hochberg (BH) FDR correction.

### Reporting summary

Further information on research design is available in the Nature Research Reporting Summary linked to this article.

## Supplementary information

Supplementary Information

Reporting Summary

## Data Availability

Sequencing data and metadata for all samples used in this study have been deposited in NCBI Sequence Read Archive (SRA) (PRJNA700829). Metabolites data on feces of miscarriage patients have been deposited in the EMBL-EBI under accession code MTBLS2465. A patient metadata file, which includes the time of sample collection and follow-up of the last pregnancies, was included in Supplementary File 1. A full record of all statistical analyses and R scripts used in this paper are included in Supplementary File 2.
