# Supplementary material for: Interactions between gut microbiota and metabolites modulate cytokine network imbalances in women with unexplained miscarriage
Source: NPJ Biofilms Microbiomes. 2021 Mar 17;7:24. doi: 10.1038/s41522-021-00199-3 (PMC7969606; doi:10.1038/s41522-021-00199-3)

Supplementary Figure S1 Flow chart describing study design and enrolment of cases and controls.

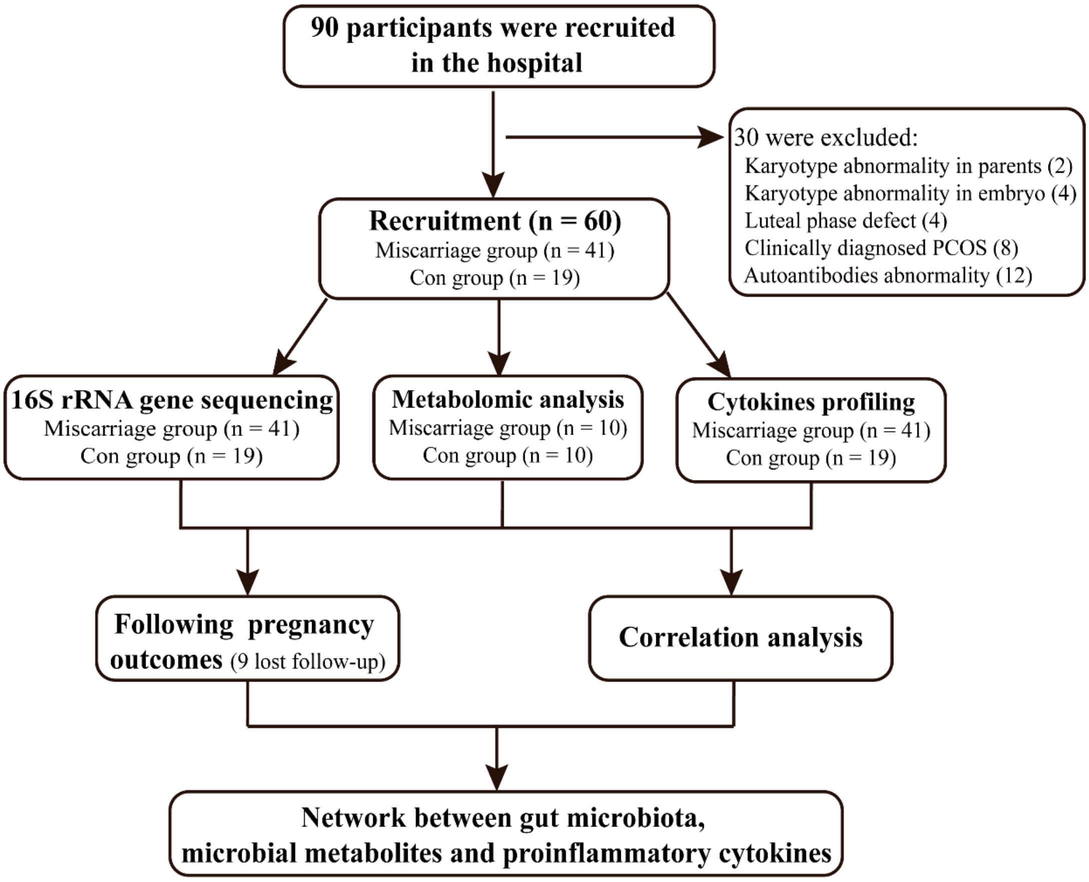

Supplementary Figure S2 Relative bacterial richness and evenness analyses. (**a**) Species accumulation curves assessing the number sequences likely required to detect additional OTUs. (**b**) Rarefaction curve evaluating the relative bacterial richness to determine whether further sequencing would identify additional OTUs.

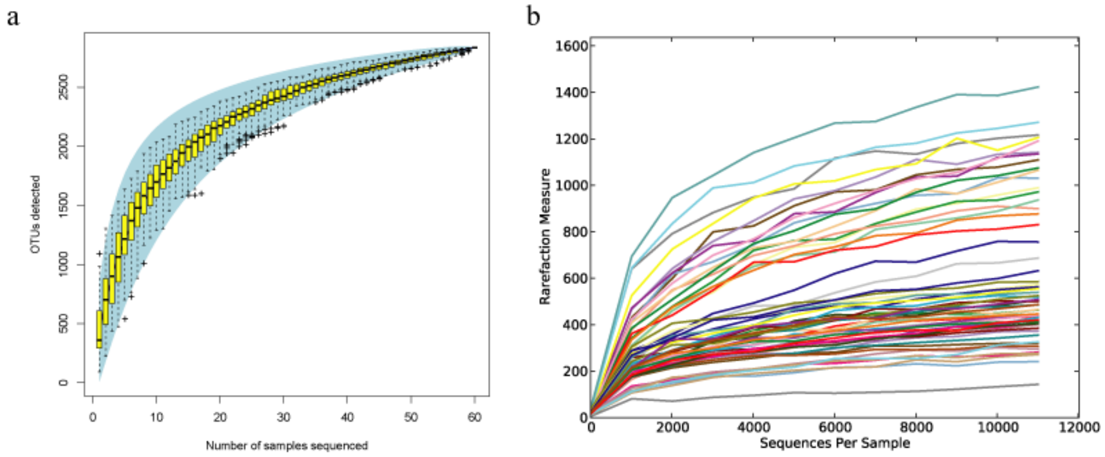

Supplementary Figure S3 Fecal microbiota profiles analyses for the metabolome subsets. (**a**) Species diversity differences between the miscarriage (n = 10) and control groups (n=10). (**b**) The top 10 significantly different genera in the relative abundances between the control and miscarriage groups; (**c**) Heatmap analyses of differentially abundant genera between the control and miscarriage groups.

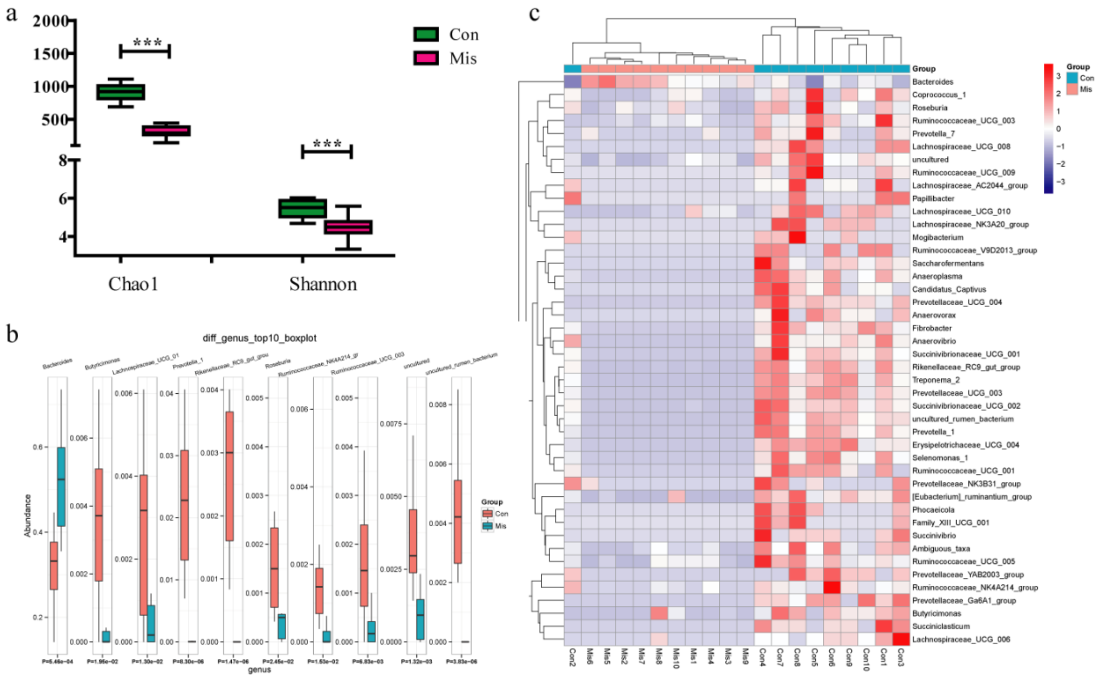

Supplementary Figure S4 Correlation analyses in bacterial genera.

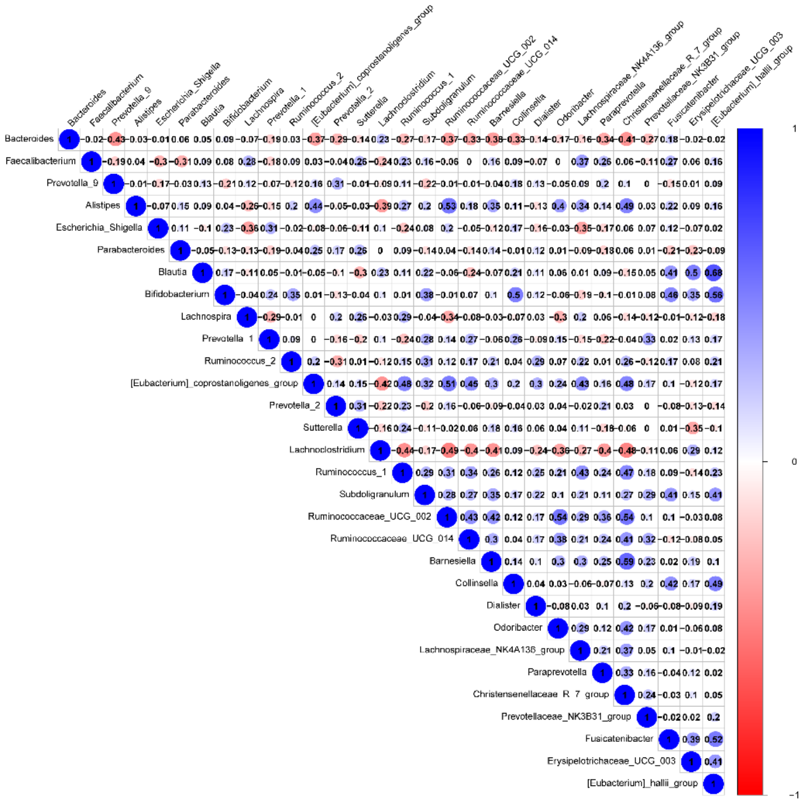

Supplementary Figure S5 Gating strategies used for cytokines detection. Gating strategies to sort the 13 cytokines including IL-2, -4, -5, -6, -9, -10, -13, -17A, -17F, -21, -22, IFN-γ, and TNF-α from human serum presented on Fig. 6a.

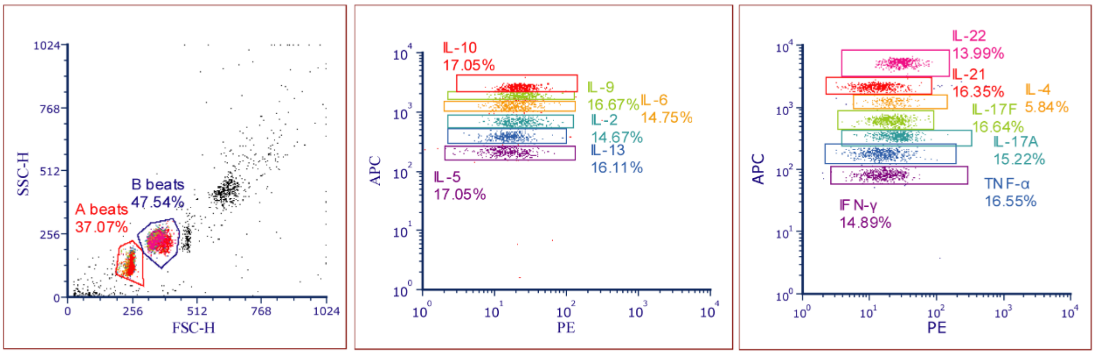

Supplementary Table S1 Genera of bacteria changed in abundance in miscarriage patients

Genera	*P*	FDR	Con	Mis_mean
Prevotellaceae_Ga6A1_group	1.30E-11	3.48E-09	0.000316	4.29E-05
Lachnospiraceae_UCG_008	1.26E-10	1.69E-08	0.000127	1.29E-05
Erysipelotrichaceae_UCG_004	3.34E-10	2.98E-08	0.001642	5.29E-05
Anaerovibrio	4.72E-10	3.17E-08	0.000588	1.29E-05
Treponema_2	6.62E-10	3.22E-08	0.00397	8.29E-04
Anaerovorax	7.20E-10	3.22E-08	0.000263	2.11E-06
Fibrobacter	1.01E-09	3.85E-08	0.000496	4.07E-06
uncultured_rumen_bacterium	1.17E-09	3.92E-08	0.008844	6.11E-06
Prevotella_1	2.34E-09	6.96E-08	0.044529	7.33E-05
Anaeroplasma	2.73E-09	7.31E-08	0.001089	4.07E-06
Saccharofermentans	3.64E-09	8.86E-08	0.000391	2.04E-06
Succinivibrionaceae_UCG_001	5.82E-09	1.30E-07	0.002147	5.07E-06
Selenomonas_1	7.26E-09	1.50E-07	0.001361	1.22E-04
Prevotellaceae_YAB2003_group	1.21E-08	2.20E-07	0.000189	6.20E-06
Prevotellaceae_UCG_003	1.23E-08	2.20E-07	0.002762	4.07E-06
Lachnospiraceae_AC2044_group	1.89E-08	3.17E-07	0.000202	1.22E-05
Succinivibrionaceae_UCG_002	9.87E-08	1.56E-06	0.003627	2.04E-06
Prevotellaceae_UCG_004	1.05E-07	1.56E-06	0.000505	5.07E-05
Succiniclasticum	1.44E-07	2.03E-06	0.000189	1.92E-05
Lachnospiraceae_NK3A20_group	1.51E-07	2.03E-06	0.000132	3.20E-05
Phocaeicola	1.65E-07	2.11E-06	9.66E-05	1.07E-06
Candidatus_Captivus	1.86E-07	2.26E-06	0.000334	1.22E-05
Ruminococcaceae_V9D2013_group	1.46E-06	1.70E-05	8.78E-05	9.81E-06
Ruminococcaceae_UCG_001	2.13E-06	2.38E-05	0.000343	1.22E-05
Haemonchus_placei	3.89E-06	4.17E-05	0.00011	2.04E-05
Ruminobacter	1.12E-05	0.000115	3.07E-05	2.07E-06
Oribacterium	1.59E-05	0.000158	0.000132	5.22E-05
Succinivibrio	1.78E-05	0.00017	0.000123	1.20E-05
Butyrivibrio_2	3.16E-05	0.000292	8.78E-05	1.07E-06
U29_B03	3.48E-05	0.000311	3.51E-05	1.22E-06
Rikenellaceae_RC9_gut_group	0.000113	0.000977	0.005722	0.000867
Lachnoclostridium_12	0.000188	0.001526	3.07E-05	8.81E-06
Lachnoclostridium_1	0.000188	0.001526	3.07E-05	8.22E-06
Anaerobiospirillum	0.000268	0.002116	6.59E-05	7.04E-06
Lachnobacterium	0.000882	0.006751	2.63E-05	2.07E-06
Papillibacter	0.001152	0.008573	9.22E-05	6.11E-06
Roseburia	0.001367	0.009904	0.002472	0.000804
Ruminiclostridium_1	0.00186	0.012534	6.59E-05	1.02E-05
Schwartzia	0.001918	0.012534	1.76E-05	9.22E-05
Lachnoclostridium_10	0.001918	0.012534	1.76E-05	9.20E-06
Candidatus_Saccharimonas	0.001918	0.012534	3.51E-05	7.81E-06
[Anaerorhabdus]_furcosa_group	0.002722	0.017367	3.07E-05	1.22E-06

probable_genus_10	0.004756	0.029642	7.90E-05	2.44E-05
Acetitomaculum	0.006909	0.042083	0.000101	2.24E-05
Pseudoramibacter	0.008462	0.048254	1.32E-05	8.10E-06
Suttonella	0.008462	0.048254	1.32E-05	7.81E-06
Spirochaeta_2	0.008462	0.048254	2.63E-05	6.22E-06
Lachnospiraceae_UCG_010	0.008867	0.04951	0.001963	0.00093

FDR, false discovery rate, represents the adjusted *P* value; Con_mean, the average abundance of genus in control group; Mis_mean, the average abundance of genus in miscarriage group; Mis, miscarriage.

Supplementary Table S2 Demographic and clinical characteristics of subjects for metabonomics

**Variables**	**Control**		***P***	**Miscarriage**		***P***
	(n = 10)	(n = 19)		(n = 10)	(n = 41)	
Age (years) [a]	30.7 ± 3.5	32.4 ± 4.7	0.25	28.3 ± 4.2	31.3 ± 5.0	0.18
Pre-pregnancy BMI (kg/m^2)	23.0 ± 2.8	22.4 ± 4.2	0.85	22.9 ± 4.0	21.5 ± 2.7	0.74
Education						
Illiterate	0 (0%) [b]	0 (0%)	NE [c]	0 (0%)	0 (0%)	NE
High school or lower	4 (40%)	4 (21%)	0.52	2 (20%)	4 (10%)	0.72
College	6 (60%)	14 (74%)	0.74	8 (80%)	31 (76%)	0.90
Postgraduate or higher	0 (0%)	1 (5%)	0.74	0 (0%)	6 (14%)	0.46
History of smoking	0 (0%)	0 (0%)	NE	0 (0%)	0 (0%)	NE
History of drinking	0 (0%)	0 (0%)	NE	0 (0%)	0 (0%)	NE
Bacterial vaginosis	0 (0%)	0 (0%)	NE	0 (0%)	0 (0%)	NE
Ureaplasma urealyticum	0 (0%)	0 (0%)	NE	0 (0%)	0 (0%)	NE
Chlamydia trachomatis	0 (0%)	0 (0%)	NE	0 (0%)	0 (0%)	NE
Trichomoniasis	0 (0%)	0 (0%)	NE	0 (0%)	0 (0%)	NE
Colpomycosis	0 (0%)	0 (0%)	NE	0 (0%)	0 (0%)	NE
Virus						
HIV+	0 (0%)	0 (0%)	NE	0 (0%)	0 (0%)	NE
HPV+	0 (0%)	0 (0%)	NE	0 (0%)	0 (0%)	NE
Syphilis	0 (0%)	0 (0%)	NE	0 (0%)	0 (0%)	NE
Antibody						
Anticardiolipin antibody (IgA, IgM, and IgG)	0 (0%)	0 (0%)	NE	0 (0%)	0 (0%)	NE
Anti DNA antibody (single and double stranded)	0 (0%)	0 (0%)	NE	0 (0%)	0 (0%)	NE
Anti ENA (Extractable nuclear antigen, 7 subtypes) antibody	0 (0%)	0 (0%)	NE	0 (0%)	0 (0%)	NE
Irregular antibody	0 (0%)	0 (0%)	NE	0 (0%)	0 (0%)	NE
Medical history						
Endometriosis	0 (0%)	0 (0%)	NE	0 (0%)	0 (0%)	NE
Uterine fibroids	0 (0%)	1 (5%)	0.74	1 (10%)	4 (8%)	0.93
Endometrial polyps	0 (0%)	0 (0%)	NE	0 (0%)	0 (0%)	NE

Intrauterine adhesion	0 (0%)	0 (0%)	NE	0 (0%)	0 (0%)	NE
Ovarian cysts	0 (0%)	0 (0%)	NE	0 (0%)	0 (0%)	NE
Pelvic inflammation	0 (0%)	0 (0%)	NE	0 (0%)	0 (0%)	NE
DUB (Dysfunctional Uterine Bleeding)	0 (0%)	0 (0%)	NE	0 (0%)	0 (0%)	NE
Rheumatoid arthritis	0 (0%)	0 (0%)	NE	0 (0%)	0 (0%)	NE
SLE(Systemic Lupus Erythematosus)	0 (0%)	0 (0%)	NE	0 (0%)	0 (0%)	NE

a, Data are presented as mean ± SD;

b, Data are presented as n (%);

c. NE, not estimable (due to nullity of category in both groups).

Supplementary Table S3 Diversity and fecal microbiota profiles comparison of the samples for metabonomics to all samples in each group in this study

**Variables**	**Control**		***P***	**Miscarriage**		***P***
	(n = 10)	(n = 19)		(n = 10)	(n = 41)	
Shannon index [a]	5.4 ±0.5	5.9 ±0.8	0.07	4.9 ±0.8	5.3 ±0.9	0.12
Chao 1 index	912 ±131	1042 ±185	0.06	407 ±108	425 ±104	0.17
Prevotella_1	0.0232	0.0445	0.12	0.0001	0.0001	0.66
Subdoligranulum	0.0128	0.0141	0.74	0.0067	0.0080	0.65
Odoribacter	0.0038	0.0118	0.14	0.0049	0.0059	0.58
Dorea	0.0062	0.0076	0.57	0.0053	0.0042	0.49
uncultured_rumen_bacterium	0.0045	0.0088	0.10	0.0000	0.0000	NE [b]
Rikenellaceae_RC9_gut_group	0.0026	0.0057	0.08	0.0000	0.0009	0.47
Lachnospiraceae_UCG_001	0.0009	0.0006	0.57	0.0004	0.0025	0.10
Roseburia	0.0018	0.0025	0.51	0.0006	0.0008	0.35
Lachnospiraceae_UCG_010	0.0027	0.0020	0.32	0.0006	0.0009	0.41
Treponema_2	0.0020	0.0040	0.09	0.0000	0.0000	NE

a, Data are presented as mean ±SD;

b. NE, not estimable (due to nullity of category in both groups).

Supplementary Table S4 Distinct metabolites in the miscarriage group compared to the control group

**Metabolites**	**VIP**	**log2(FC)**	***P*-value**	**FDR**
Hyocholic acid	33.70	1.76	3.09E-02	0.48
Methyl dihydrophaseate	32.77	3.12	3.82E-03	0.31
Pregnan-20-one, 17-(acetyloxy)-3-	32.50	-1.63	4.85E-03	0.31
3a,7a,12b-Trihydroxy-5b-cholanoic acid	27.74	1.52	4.00E-02	0.51
3-keto Petromyzonol	26.73	-1.26	2.90E-02	0.47
Hyodeoxycholic acid	26.50	-1.19	2.31E-02	0.45
3a,6a,7b-Trihydroxy-5b-cholanoic acid	20.13	1.85	2.35E-02	0.45
3alpha-Hydroxy-5beta-chola-8,14-dien-24-oic Acid	19.92	1.52	2.14E-02	0.45
3,8-Dihydroxy-6-methoxy-7(11)-eremophilen-12,8-o	18.57	2.84	3.37E-03	0.30
D-Urobilinogen	18.12	1.49	5.64E-03	0.33
7-Hydroxy-3-oxocholanoic acid	17.30	-1.78	1.29E-03	0.26
3alpha-Hydroxy-5beta-chola-8(14),11-dien-24-oic A	15.96	1.70	9.63E-03	0.37
1b,3a,7b-Trihydroxy-5b-cholanoic acid	12.78	1.46	2.80E-02	0.47
THA	12.07	1.10	3.59E-02	0.50
Isolithocholic acid	11.67	-1.68	1.98E-02	0.44
Chenodeoxycholic acid sulfate	10.54	1.07	4.10E-02	0.51
3β-Hydroxychola-5,7-dien-24-oic Acid	9.21	1.92	7.48E-03	0.35
Lucidenic acid J	8.79	1.57	1.84E-02	0.43
prasterone sulfate	8.26	1.26	2.42E-03	0.29
Ursocholic acid	6.93	1.63	3.30E-02	0.49
7alpha,12alpha-Dihydroxy-5beta-chol-3-en-24-oic A	6.11	1.53	4.33E-02	0.52
Adrenic acid	5.76	1.08	2.69E-03	0.29
PI(18:3(6Z,9Z,12Z)/16:0)	5.52	3.58	5.93E-03	0.33
L-isoleucyl-L-proline	5.13	2.12	9.78E-03	0.38
PI(15:0/20:3(8Z,11Z,14Z))	4.91	2.28	1.17E-02	0.40
Deoxycholic acid 3-glucuronide	4.88	1.15	1.68E-02	0.42
Mequitazine	4.27	0.93	5.70E-03	0.33
Malyngamide T	4.19	2.24	2.17E-03	0.28
2-Piperidinone	4.15	0.97	3.51E-02	0.49
CUDA	4.14	-1.45	2.19E-02	0.45
PI(P-16:0/18:1(9Z))	4.03	-1.95	2.20E-04	0.18
1-L-Leucyl-L-Proline	4.00	1.95	1.12E-02	0.39
4alpha-Carboxy-4beta-methyl-5alpha-cholesta-8-en	3.96	-2.53	2.93E-02	0.47
11-acetoxy-3beta,6alpha-dihydroxy-24-methyl-27-n	3.89	2.16	2.45E-02	0.46
Cortisone	3.75	-1.41	8.92E-03	0.37
PI(O-18:0/16:0)	3.72	-1.04	3.98E-02	0.51
Menatetrenone	3.68	-0.68	3.04E-02	0.48
Rodiasine	3.68	1.42	1.19E-03	0.26
Isogingerenone B	3.56	-0.73	4.15E-02	0.51
7a-Hydroxydehydroepiandrosterone	3.46	1.32	1.22E-02	0.40
Oxidanesulfonic acid	3.28	6.41	3.30E-02	0.49

Coagulin R 3-glucoside	3.27	2.46	5.70E-04	0.23
Norcodeine	3.25	2.40	8.40E-04	0.25
(+)-Tetrandrine	3.24	0.99	4.53E-03	0.31
Camelliagenin C	3.18	-0.82	3.76E-02	0.50
2-Hydroxychrysophanol	3.09	3.59	3.91E-02	0.50
CARAPIN	3.01	1.03	9.20E-03	0.37
Hydromorphone	2.99	1.05	6.06E-03	0.33
Timolol	2.96	0.82	1.60E-04	0.18
PG(a-13:0/i-24:0)	2.96	-1.74	2.15E-03	0.28
Acetylcholine	2.95	1.30	3.63E-02	0.50
Digoxigenin monodigitoxoside	2.92	1.67	2.21E-02	0.45
(2E)-Piperamide-C5:1	2.90	2.26	4.59E-03	0.31
DL-β-Leucine	2.90	-2.85	1.26E-03	0.26
1-hexadecanoyl-2-octadecanoyl-sn-glycero-3-phos	2.85	2.54	3.41E-03	0.30
CL(8:0/8:0/8:0/16:0)	2.84	2.73	4.89E-02	0.52
N-arachidonoyl isoleucine	2.79	-1.34	1.84E-02	0.43
3-(3-hydroxyphenyl)-2-phenyl-4-[(E)-2-phenyletheny	2.78	1.59	1.30E-06	0.01
(±)-Ribaline	2.75	2.82	4.38E-02	0.52
3-(4-hydroxyphenyl)-3,4-dihydro-2H-1-benzopyran-	2.70	-3.32	1.46E-02	0.42
Glyinflanin C	2.68	1.46	8.20E-06	0.03
Scilliroside	2.64	-2.36	3.52E-02	0.49
3beta,6beta-Dihydroxynortropane	2.61	3.90	4.44E-03	0.31
13-HDoHE	2.55	-1.43	4.56E-03	0.31
1,4-Methylimidazoleacetic acid	2.51	1.48	1.20E-04	0.18
DG(15:0/18:3(6Z,9Z,12Z)/0:0)	2.49	-1.57	2.56E-03	0.29
PI(20:0/14:0)	2.45	2.70	1.65E-02	0.42
PI(P-18:0/0:0)	2.44	0.63	1.32E-02	0.41
Anhydroamarouciaxanthin B	2.42	-0.74	1.13E-02	0.39
PtdIns-(1,2-dioctanoyl) (sodium salt)	2.39	1.45	2.34E-02	0.45
3,6-dihydroxy-4,5-diphenyl-2,3,4,5-tetrahydropyridin	2.39	1.43	1.60E-02	0.42
3-hydroxyarachidonoylcarnitine	2.38	1.40	4.56E-02	0.52
1-Nitrohexane	2.37	1.79	2.00E-04	0.18
N-[(3a,5b,7a)-3-hydroxy-24-oxo-7-(sulfooxy)cholan-	2.35	1.91	3.98E-02	0.51
PC(P-17:0/0:0)	2.34	-0.93	3.54E-03	0.30
VPGPR Enterostatin	2.32	-0.82	4.11E-02	0.51
PC(O-16:0/0:0)	2.31	-0.65	1.57E-02	0.42
Megalomicin C2	2.26	1.66	6.44E-03	0.34
12-Oxo-20-carboxy-leukotriene B4	2.25	1.13	6.19E-03	0.34
4,4-Dimethylcholesta-8,14,24-trienol	2.25	-2.65	9.49E-03	0.37
2,4,6-octatrienal	2.20	-2.38	4.69E-02	0.52
DG(14:0/0:0/14:0) (d5)	2.16	-0.41	1.03E-02	0.38
2-bromopalmitaldehyde	2.15	0.69	4.64E-02	0.52
Isodomoic acid A	2.14	1.53	5.94E-03	0.33
Avideoxycholic acid	2.14	-0.82	3.89E-02	0.50

Nonanoylcarnitine	2.13	4.83	3.18E-02	0.48
Tamoxifen	2.02	2.32	3.54E-02	0.49
Lysyl-Gamma-glutamate	2.00	-0.84	1.51E-02	0.42
15-HETE-DA	1.99	-1.17	3.69E-02	0.50
Butabarbital	1.99	1.08	3.68E-02	0.50
Phorbol	1.95	-0.72	1.97E-02	0.44
PI(P-20:0/18:4(6Z,9Z,12Z,15Z))	1.95	1.30	4.04E-02	0.51
stigmasta-7,22E,25-trien-3β-ol	1.94	-1.75	5.97E-03	0.33
keratan sulfate I	1.94	1.15	4.63E-03	0.31
(S)-(-)-Acenocoumarol	1.94	4.81	3.71E-02	0.50
Tyrosyl-Lysine	1.90	-1.48	4.70E-02	0.52
omega-hydroxyfinasteride	1.90	0.82	1.32E-02	0.41
forskolin	1.84	1.27	1.47E-03	0.27
PI(12:0/20:4(5Z,8Z,11Z,14Z))	1.84	-1.51	2.80E-04	0.20
12(S)-HETE	1.83	-1.78	4.36E-02	0.52
Domoic acid	1.83	1.46	8.38E-03	0.36
C16 Sphinganine	1.82	-0.62	4.96E-02	0.52
L-Carnitine	1.82	1.21	4.50E-02	0.52
Clausarinol	1.81	-1.14	1.60E-03	0.27
(R)-1-O-[b-D-Glucopyranosyl-(1->6)-b-D-glucopyra	1.79	0.96	1.07E-02	0.39
Deoxyinosine	1.79	0.98	3.62E-02	0.50
mLPA(O-16:0)	1.75	-0.92	3.79E-02	0.50
PI(P-18:0/20:4(5Z,8Z,11Z,14Z))	1.71	2.29	1.61E-02	0.42
Isolinderanolide	1.70	-2.27	4.36E-02	0.52
7-undecenoic acid	1.67	-2.14	1.33E-02	0.41
Sphingofungin F	1.66	3.20	2.32E-02	0.45
3-Methyl-2-butenoic acid	1.66	0.98	3.74E-02	0.50
Polyporusterone F	1.65	-0.67	4.89E-02	0.52
(-)-Fumigaclavine B	1.64	1.03	1.20E-02	0.40
1alpha-hydroxy-24-(dimethoxyphosphoryl)-25,26,27	1.63	1.62	2.19E-02	0.45
Hydrocinnamic acid	1.61	-2.16	2.46E-02	0.46
5alpha-androstane-3alpha-ol-17-one sulfate	1.61	0.88	4.83E-02	0.52
Terretonin	1.61	3.24	3.31E-02	0.49
24-ketocholestanol	1.59	-1.49	2.29E-02	0.45
2,3-Dinor-11b-PGF2a	1.59	-0.74	4.48E-03	0.31
(S)-a-Amino-2,5-dihydro-5-oxo-4-isoxazolepropanoi	1.59	1.26	6.96E-03	0.34
Avermectin A2a	1.58	1.21	2.36E-03	0.29
Pyroglutamic acid	1.53	1.07	4.95E-02	0.52
(22S)-22-hydroxyvitamin D3	1.53	0.57	2.16E-02	0.45
7-Methyl-1,4,5-naphthalenetriol 4-[xylosyl-(1->6)-glu	1.53	-0.88	4.10E-02	0.51
Heptadecanoyl carnitine	1.52	-0.72	1.17E-02	0.40
19-hydroxy-17-oxoandrost-5-en-3-β-yl sulfate	1.52	0.46	4.16E-02	0.51
PI(18:3(9Z,12Z,15Z)/14:1(9Z))	1.52	-1.55	1.19E-03	0.26
Paxilline	1.50	-1.36	7.03E-03	0.34

12-amino-octadecanoic acid	1.49	1.51	7.66E-03	0.35
5-Methoxy-7-(4-hydroxyphenyl)-1-phenyl-3-heptano	1.48	-2.05	4.14E-02	0.51
Gibberellin GA126	1.45	-0.68	3.46E-02	0.49
Allyl tiglate	1.44	1.09	3.15E-02	0.48
Eugenyl formate	1.44	1.72	4.10E-04	0.21
PE-Cer(d14:1(4E)/20:1(11Z)(2OH))	1.43	-1.39	2.90E-02	0.47
alpha-Tocopherol succinate	1.43	-0.32	2.53E-03	0.29
Arecoline	1.42	1.80	2.16E-02	0.45
(6beta,22E)-6-Hydroxystigmasta-4,22-dien-3-one	1.42	-4.05	3.79E-02	0.50
O-Desmethylangolensin	1.42	-4.73	1.38E-02	0.41
Amdinocillin	1.41	-2.01	2.36E-02	0.45
Naftifine	1.41	0.85	9.09E-03	0.37
PGD2-dihydroxypropanylamine	1.40	0.90	4.76E-02	0.52
Phloretin xylosyl-galactoside	1.40	2.43	3.39E-02	0.49
PGP(16:0/18:0)	1.40	1.45	1.67E-02	0.42
Dichotosin	1.39	0.60	3.58E-02	0.50
Ganosporelactone A	1.39	-3.01	3.93E-02	0.50
Alliogenin	1.38	0.40	2.36E-02	0.45
20-acetoxy-clavulone III	1.38	-1.43	2.55E-02	0.46
Sebacic acid	1.38	-0.81	3.25E-02	0.48
Lucidenic acid G	1.38	0.89	3.76E-02	0.50
alpha,beta-Dihydroxanthohumol	1.37	-1.23	4.33E-03	0.31
5-(1-oxopropan-2-yl)isolongifol-5-ene	1.37	-1.09	4.51E-02	0.52
Nitramine	1.37	-2.14	2.48E-02	0.46
(S)-Reticuline	1.37	-0.83	4.14E-03	0.31
PS(20:4(5Z,8Z,11Z,14Z)/22:6(4Z,7Z,10Z,13Z,16Z,19	1.36	-1.50	3.80E-04	0.21
16,16-dimethyl-6-keto Prostaglandin E1	1.36	-0.58	4.32E-02	0.52
(S)-N-Methylcoclaurine	1.35	0.98	9.70E-04	0.25
Biotripyrrin-b	1.33	1.12	1.68E-02	0.42
Ergothioneine	1.33	0.55	4.85E-02	0.52
Dodecanedioic acid	1.33	-1.42	1.66E-02	0.42
7beta-Hydroxy-lathyrol	1.32	0.34	4.49E-02	0.52
8-Prenyllepidissipyrone	1.32	-1.65	1.04E-03	0.25
(1(10)E,4a,5E)-1(10),5-Germacradiene-12-acetoxy-	1.30	-0.66	8.81E-03	0.37
Gamma glutamyl ornithine	1.30	2.03	4.84E-03	0.31
16-F1-PhytoP	1.30	-0.80	9.49E-03	0.37
Physagulin G	1.30	0.80	7.20E-04	0.24
Dynorphin B (10-13)	1.30	2.31	2.37E-02	0.45
1-heptadecanoyl-glycero-3-phosphate	1.29	1.05	1.78E-02	0.43
Withanolide A	1.29	0.83	7.15E-03	0.34
11-dehydro-2,3-dinor-TXB2	1.26	-0.77	2.14E-02	0.45
Zedoarondiol	1.26	-0.63	1.31E-02	0.41
15-Deoxy-d-12,14-PGJ2	1.26	-0.97	5.30E-03	0.32
L-threo-Sphingosine C-18	1.25	-1.81	3.99E-02	0.51

Voglibose	1.25	1.07	1.65E-03	0.27
Zanthodioline	1.25	0.62	2.54E-02	0.46
6-Hydroxyenterolactone	1.24	-0.83	2.70E-02	0.46
(1S,16R)-5,7,11-trihydroxy-3-	1.24	1.44	3.82E-02	0.50
16,16-dimethyl-PGE1	1.24	-0.71	4.81E-03	0.31
Photobarbatusin I	1.23	0.41	6.28E-03	0.34
2-(1-Ethoxyethoxy)propanoic acid	1.23	2.86	3.22E-02	0.48
4,5-dihydroxy-2-	1.23	7.82	4.68E-02	0.52
Quifenadine	1.23	0.51	2.13E-02	0.45
Thymidine	1.22	1.09	4.11E-02	0.51
(1R,12S,16Z,24E,26E,28Z,32S)-1,18-dihydroxy-12-	1.22	2.83	6.50E-04	0.23
9,12-dioxo-dodecanoic acid	1.22	-0.85	3.31E-02	0.49
Bepridil	1.21	-1.70	1.47E-03	0.27
Hydrocortisone caproate	1.19	-0.63	2.19E-02	0.45
N-oleoyl histidine	1.19	-1.58	6.85E-03	0.34
Dolastatin 16	1.19	1.31	4.53E-02	0.52
4-Hydroxybenzyl isothiocyanate 4''-acetylrhamnosid	1.19	6.50	3.87E-02	0.50
15-keto Latanoprost (free acid)	1.18	0.71	4.99E-02	0.52
Malvidin	1.18	0.66	1.89E-02	0.43
TXB2	1.18	-0.77	1.06E-02	0.39
13,14-dihydro-16,16-difluoro Prostaglandin D2	1.17	-0.87	8.37E-03	0.36
19(R)-hydroxy-PGF1α	1.17	-0.74	2.27E-02	0.45
Exiguaflavanone E	1.17	0.89	2.26E-02	0.45
11-[(2R,3S)-3-[2-amino-3-methyl-4-	1.17	-0.92	2.89E-02	0.47
Nordihydrocapsiate	1.17	-1.24	4.13E-02	0.51
PF-750	1.16	0.55	3.18E-02	0.48
PI(18:2(9Z,12Z)/17:1(9Z))	1.15	1.87	2.23E-02	0.45
7,12-Dimethylbenz[a]anthracene	1.15	1.05	4.60E-03	0.31
Decanoylcarnitine	1.15	1.56	1.34E-02	0.41
LysoPC(22:1(13Z))	1.15	1.06	1.43E-02	0.41
Nandrolone	1.15	1.55	3.96E-02	0.50
Tuftsin	1.14	0.53	1.60E-02	0.42
Candoxatril	1.14	2.49	3.01E-02	0.48
Laurotetanine	1.13	0.48	2.87E-03	0.29
Arenaine	1.13	0.65	2.43E-02	0.46
PC(16:0/5:1(4E))	1.12	-1.03	1.12E-02	0.39
Fluvoxamine	1.12	-1.34	1.58E-02	0.42
(3b,6b,8a,12a)-8,12-Epoxy-7(11)-eremophilene-	1.11	-0.95	2.61E-02	0.46
Homohyocholic acid	1.11	1.43	3.42E-02	0.49
PI(22:6(4Z,7Z,10Z,13Z,16Z,19Z)/0:0)	1.10	0.76	5.14E-03	0.32
2-Amino-5-oxohexanoate	1.10	1.35	3.04E-02	0.48
1alpha,25-dihydroxy-21-nor-20-oxavitamin D3	1.10	-1.39	8.84E-03	0.37
Dehydroepiandrosterone sulfate	1.09	1.27	3.59E-03	0.30
Mycinamicin IV	1.09	-1.10	2.53E-02	0.46

MGDG-O(16:3(7Z,10Z,13Z))	1.08	1.62	3.45E-03	0.30
6-{[8-(2,4-dihydroxyphenyl)-7-	1.08	1.85	1.44E-02	0.42
Kessyl glycol	1.07	-2.56	1.74E-02	0.42
alpha-dihydrotetrabenazine	1.07	1.08	1.39E-02	0.41
Valganciclovir	1.06	-1.49	1.79E-02	0.43
Bisnorcholic acid	1.06	-0.89	2.88E-02	0.47
sn-3-O-(geranylgeranyl)glycerol 1-phosphate	1.06	-0.96	3.50E-02	0.49
Allose	1.05	0.49	4.89E-02	0.52
Crustecdysone	1.05	-0.48	1.78E-02	0.43
3-pentadecylphenol	1.05	-1.69	1.71E-02	0.42
1,24-Dihydroxy-25-fluorovitamin D3	1.04	-0.68	1.06E-03	0.25
Cerbertin	1.04	0.45	1.35E-02	0.41
MK 0457	1.04	0.20	2.71E-02	0.46
Mirtazapine	1.04	0.77	1.36E-02	0.41
Imidazolepropionic acid	1.04	1.94	1.20E-04	0.18
N-linolenoyl glutamine	1.03	-0.54	5.87E-03	0.33
3-hydroxy-3-methyl-Glutaric acid	1.03	0.63	3.08E-02	0.48
6-beta-hydroxydexamethasone	1.02	-0.55	1.39E-03	0.27
Isorumelenic acid	1.01	-1.14	2.30E-02	0.45
(9R,13R)-1a,1b-dinor-10,11-dihydro-12-oxo-	1.01	-0.76	2.30E-02	0.45
19(R)-hydroxy-PGE1	1.01	-0.63	3.11E-02	0.48
4-(3-Hydroxy-7-phenyl-6-heptenyl)-1,2-benzenediol	1.00	2.08	1.24E-03	0.26

VIP, variable importance in the projection; FC, fold change.

Supplementary File 1 Metadata associated with all samples used in this study.

Sample name	Source	Organism	Nation	Phenotype	Gestational weeks	Sampling time	Sampling site	Time of follow-up:	Outcome of the following pregnancy
Mis1	Feces	*Homo sapiens*	Han Chinese	Miscarriage	8	Aug-17	at home	Dec-19	vaginal delivery
Mis2	Feces	*Homo sapiens*	Han Chinese	Miscarriage	8	Aug-17	hospital	Dec-19	non successful pregnant
Mis3	Feces	*Homo sapiens*	Han Chinese	Miscarriage	8	Sep-17	at home	Dec-19	vaginal delivery
Mis4	Feces	*Homo sapiens*	Han Chinese	Miscarriage	8	Aug-17	hospital	Dec-19	non successful pregnant
Mis5	Feces	*Homo sapiens*	Han Chinese	Miscarriage	8	Sep-17	at home	Dec-19	contraception
Mis6	Feces	*Homo sapiens*	Han Chinese	Miscarriage	8	Sep-17	at home	Dec-19	non successful pregnant
Mis7	Feces	*Homo sapiens*	Han Chinese	Miscarriage	8	Sep-17	at home	Dec-19	cesarean section
Mis8	Feces	*Homo sapiens*	Han Chinese	Miscarriage	6	Oct-17	hospital	Dec-19	lost follow-up
Mis9	Feces	*Homo sapiens*	Han Chinese	Miscarriage	8	Sep-17	at home	Dec-19	cesarean section
Mis10	Feces	*Homo sapiens*	Han Chinese	Miscarriage	8	Oct-17	at home	Dec-19	vaginal delivery
Mis11	Feces	*Homo sapiens*	Han Chinese	Miscarriage	8	Oct-17	hospital	Dec-19	cesarean section
Mis12	Feces	*Homo sapiens*	Han Chinese	Miscarriage	8	Oct-17	at home	Dec-19	lost follow-up
Mis13	Feces	*Homo sapiens*	Han Chinese	Miscarriage	8	Oct-17	hospital	Dec-19	non successful pregnant
Mis14	Feces	*Homo sapiens*	Han Chinese	Miscarriage	8	Sep-17	at home	Dec-19	contraception
Mis15	Feces	*Homo sapiens*	Han Chinese	Miscarriage	8	Sep-17	hospital	Dec-19	vaginal delivery
Mis16	Feces	*Homo sapiens*	Han Chinese	Miscarriage	12	Oct-17	at home	Dec-19	vaginal delivery
Mis17	Feces	*Homo sapiens*	Han Chinese	Miscarriage	8	Oct-17	hospital	Dec-19	cesarean section
Mis18	Feces	*Homo sapiens*	Han Chinese	Miscarriage	8	Sep-17	at home	Dec-19	contraception
Mis19	Feces	*Homo sapiens*	Han Chinese	Miscarriage	8	Oct-17	at home	Dec-19	non successful pregnant
Mis20	Feces	*Homo sapiens*	Han Chinese	Miscarriage	8	Nov-17	hospital	Dec-19	pregnancy loss

Mis21	Feces	*Homo sapiens*	Han Chinese	Miscarriage	8	Aug-17	at home	Dec-19	non successful pregnant
Mis22	Feces	*Homo sapiens*	Han Chinese	Miscarriage	8	Dec-17	at home	Dec-19	vaginal delivery
Mis23	Feces	*Homo sapiens*	Han Chinese	Miscarriage	8	Nov-17	at home	Dec-19	vaginal delivery
Mis24	Feces	*Homo sapiens*	Han Chinese	Miscarriage	8	Nov-17	hospital	Dec-19	contraception
Mis25	Feces	*Homo sapiens*	Han Chinese	Miscarriage	8	Sep-17	at home	Dec-19	non successful pregnant
Mis26	Feces	*Homo sapiens*	Han Chinese	Miscarriage	8	Dec-17	hospital	Dec-19	pregnancy loss
Mis27	Feces	*Homo sapiens*	Han Chinese	Miscarriage	12	Aug-17	at home	Dec-19	non successful pregnant
Mis28	Feces	*Homo sapiens*	Han Chinese	Miscarriage	8	Dec-17	hospital	Dec-19	non successful pregnant
Mis29	Feces	*Homo sapiens*	Han Chinese	Miscarriage	8	Sep-17	at home	Dec-19	non successful pregnant
Mis30	Feces	*Homo sapiens*	Han Chinese	Miscarriage	8	Oct-17	at home	Dec-19	vaginal delivery
Mis31	Feces	*Homo sapiens*	Han Chinese	Miscarriage	8	Dec-17	at home	Jan-20	vaginal delivery
Mis32	Feces	*Homo sapiens*	Han Chinese	Miscarriage	12	Oct-17	at home	Dec-19	non successful pregnant
Mis33	Feces	*Homo sapiens*	Han Chinese	Miscarriage	8	Oct-17	at home	Dec-19	contraception
Mis34	Feces	*Homo sapiens*	Han Chinese	Miscarriage	8	Nov-17	at home	Jan-20	vaginal delivery
Mis35	Feces	*Homo sapiens*	Han Chinese	Miscarriage	8	Jan-18	hospital	Dec-19	pregnancy loss
Mis36	Feces	*Homo sapiens*	Han Chinese	Miscarriage	8	Mar-18	at home	Dec-19	lost follow-up
Mis37	Feces	*Homo sapiens*	Han Chinese	Miscarriage	8	Jan-18	hospital	Dec-19	lost follow-up
Mis38	Feces	*Homo sapiens*	Han Chinese	Miscarriage	8	Sep-17	at home	Jan-20	vaginal delivery
Mis39	Feces	*Homo sapiens*	Han Chinese	Miscarriage	8	Nov-17	hospital	Dec-19	cesarean section
Mis40	Feces	*Homo sapiens*	Han Chinese	Miscarriage	8	Jan-18	at home	Dec-19	lost follow-up
Mis41	Feces	*Homo sapiens*	Han Chinese	Miscarriage	8	Feb-18	at home	Dec-19	lost follow-up
Con1	Feces	*Homo sapiens*	Han Chinese	Control	8	Aug-17	at home	Dec-19	contraception

Con2	Feces	*Homo sapiens*	Han Chinese	Control	8	Oct-17	at home	Dec-19	contraception
Con3	Feces	*Homo sapiens*	Han Chinese	Control	8	Oct-17	at home	Dec-19	contraception
Con4	Feces	*Homo sapiens*	Han Chinese	Control	8	Oct-17	at home	Dec-19	lost follow-up
Con5	Feces	*Homo sapiens*	Han Chinese	Control	8	Nov-17	at home	Dec-19	contraception
Con6	Feces	*Homo sapiens*	Han Chinese	Control	8	Dec-17	hospital	Dec-19	pregnancy loss
Con7	Feces	*Homo sapiens*	Han Chinese	Control	8	Nov-17	at home	Dec-19	contraception
Con8	Feces	*Homo sapiens*	Han Chinese	Control	8	Dec-17	hospital	Dec-19	contraception
Con9	Feces	*Homo sapiens*	Han Chinese	Control	8	Nov-17	at home	Dec-19	lost follow-up
Con10	Feces	*Homo sapiens*	Han Chinese	Control	8	Dec-17	at home	Dec-19	contraception
Con11	Feces	*Homo sapiens*	Han Chinese	Control	8	Jun-18	at home	Dec-19	non successful pregnant
Con12	Feces	*Homo sapiens*	Han Chinese	Control	8	Jun-18	at home	Dec-19	contraception
Con13	Feces	*Homo sapiens*	Han Chinese	Control	8	Jul-18	hospital	Dec-19	lost follow-up
Con14	Feces	*Homo sapiens*	Han Chinese	Control	8	Jan-18	at home	Dec-19	contraception
Con15	Feces	*Homo sapiens*	Han Chinese	Control	8	Jul-18	at home	Dec-19	non successful pregnant
Con16	Feces	*Homo sapiens*	Han Chinese	Control	8	Jun-18	hospital	Dec-19	contraception
Con17	Feces	*Homo sapiens*	Han Chinese	Control	8	Mar-18	at home	Dec-19	contraception
Con18	Feces	*Homo sapiens*	Han Chinese	Control	8	Mar-18	at home	Dec-19	contraception
Con19	Feces	*Homo sapiens*	Han Chinese	Control	8	Oct-18	hospital	Dec-19	contraception

Supplementary File 2 Full account of statistical analysis performed in R.

This document contains all statistical analyses conducted for the manuscript. Note that due to the random iterative nature of some analyses (such as RDA & PCA) some of the figure parameters will change slightly during reanalysis, though core results will remain essentially unchanged.

Results:

PCoA:

```
# Qiime software
multiple_rarefactions_even_depth.py -i otu_biom -d min_depth -n 10 -o output
--lineages_included
beta_diversity.py -i output -m
weighted_unifrac,unweighted_unifrac,bray_curtis,binary_jaccard,euclidean -o
beta_diversity -t min_depth
make_emperor.py                                                        –I
beta_diversity/unweighted_unifrac_pcoa_coordinates_pcoa_coordinates
-m mapping.txt -o beta_diversity/unweighted_unifrac_pcoa_jackknifed_3D_PCoA
```

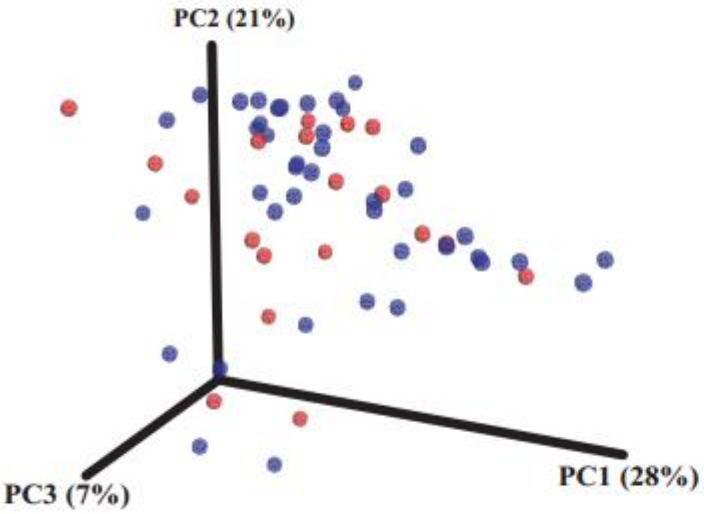

Boxplot:

```
library(ggplot2)
a<-read.table('diff_box.txt',sep="\t",header=T)
group = length(unique(a$Group))
a$P<-as.factor(a$P)
fa<- unique(a$Group)
a$Group<-factor(a$Group,levels=fa)
ggplot(data=a, aes(x=axis,y=Abundance))+geom_boxplot(aes(fill=Group),outlier.size
= 0)+
facet_wrap( ~ id, scales="free",nrow=1)+
```

labs(title=newname,x='genus',y="Abundance")+theme_bw()+
theme(axis.text.x = element_text(size=8,angle = 0,face = "bold",vjust=0),strip.text.x = element_text(colour = "black", angle = 15, size = 8,hjust = 0.5, vjust = 0.4),strip.background = element_rect(colour = "white", fill = "white"))
ggsave('diff_box_top10.pdf',width=7,limitsize=FALSE)

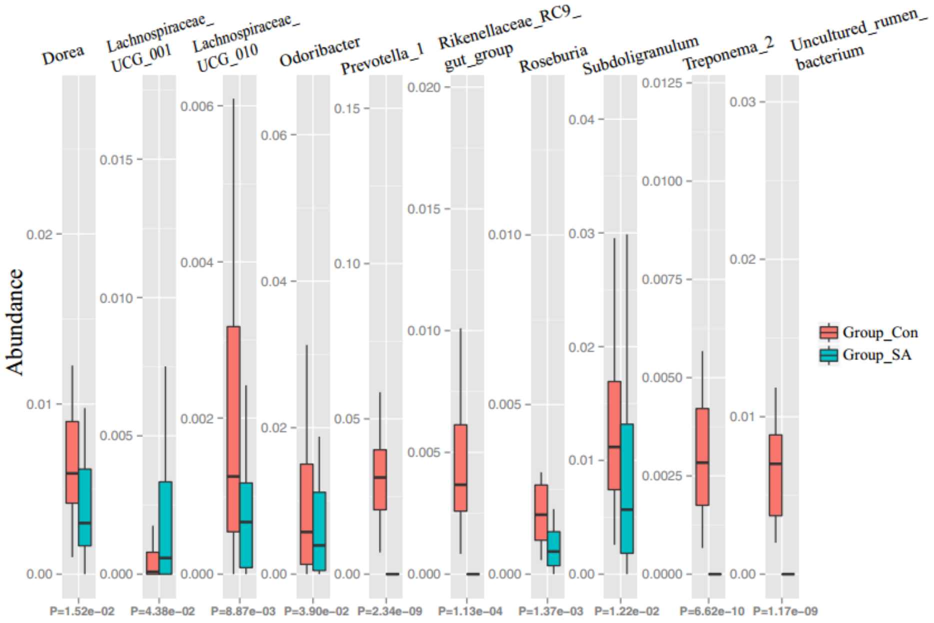

LEfSe:
code download from http://huttenhower.sph.harvard.edu/galaxy/
Fig3A
python plot_res.py LEfSe_table.res --left_space 0.3 --right_space 0.3 LEfSe_table.res.pdf --format pdf
Fig3B
python plot_cladogram.py LEfSe_table.res LEfSe_table.cladogram-add_genuslabel.pdf --format pdf --labeled_stop_lev 6 --abrv_stop_lev 6 --title_font_size 9 --label_font_size 4 --class_legend_font_size 5 --right_space_prop 0.32

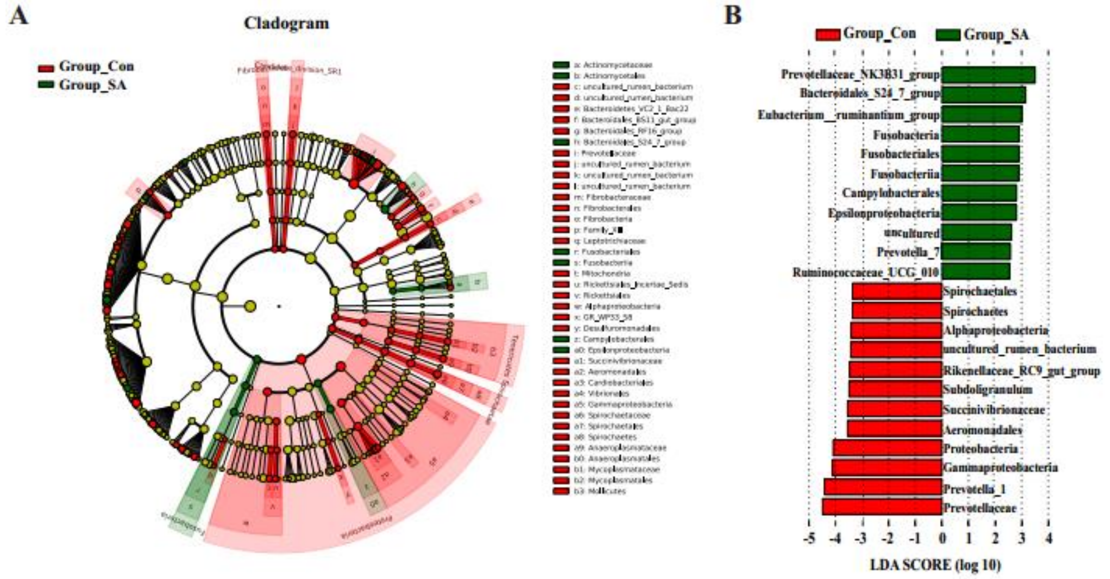

OPLS-DA:
software:SIMCA 14.1

Volcano plot:

```
library("ggplot2")
data<- read.table("vol.txt",head = T,sep="/t",row.names=1,encoding="utf-
8",check.names=F,quote="",as.is=TRUE)
ggplot(data, aes(x= FC, y= P))+
geom_point(aes(color=Status))+
scale_color_manual(values=c("blue","grey","red"))+
labs(title="Volcanoplot",x="log2(FC)",y="-log10(pvalue)")+
geom_hline(yintercept=1.3,linetype=2)+theme_bw()+theme(panel.grid=element_blan
k(),legen
d.position=c(0.9,0.9),plot.title = element_text(size = 15,face = "bold", vjust = 0.5,
hjust = 0.5),legend.background =
ggplot2::element_rect(fill="white",colour="black",size=0.3),legend.margin =
margin(t =
0, r = 6, b = 6, l = 2, unit = "pt"))+geom_hline(yintercept = c(-
log10(0.05)),linetype="dashed",size=0.5,colour = "black")+
guides(color=guide_legend(title=NULL))+
annotate("text", label = paste0("p-value=",0.05), x = 8, y = c(-log10(0.05))-0.2,
size = 4, colour = "black")
```

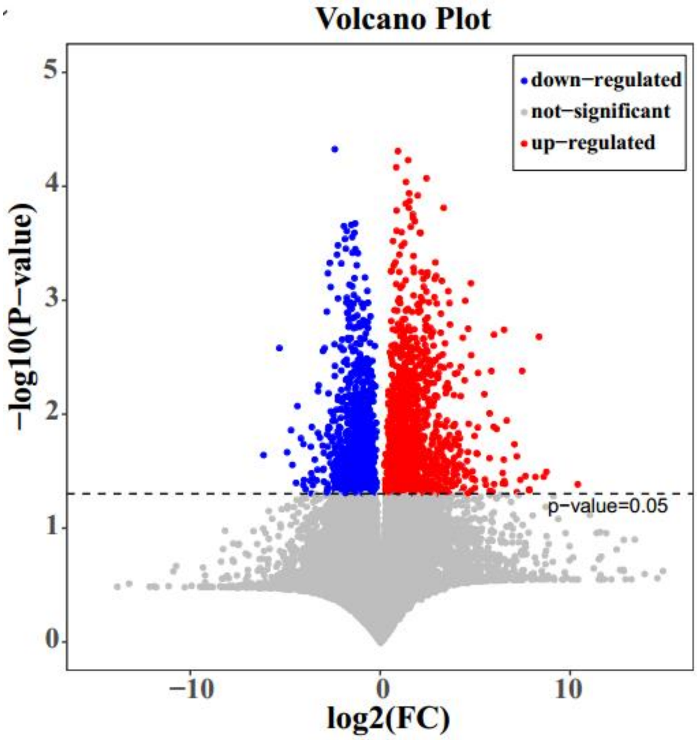

Barplot:

```
data<- read.table("rich.txt",head = T,sep="/t",row.names=1,encoding="utf-
8",check.names=F,quote="",as.is=TRUE)
cex=4.8/(2+sqrt(dim(data)[1]))
colwidth=c(0.01,rep(0.5,dim(data)[1]),0.01)
ylab="-log10(p-value)"
space=1+2/dim(data)[1]
col=c("#FFFFFF",colorRampPalette(c("black", "blue"))(dim(data)[1]),"#FFFFFF")
srt=40
family="sans"
opar <- par(no.readonly = TRUE)
par(mar =c(18,15,5,5),font.lab=2)
labels <- as.vector(data[,1])
labels1<-c("",labels,"")
counts <- as.vector(data[,2])
counts1 <- c(0,counts,0)
if((max(counts)-floor(max(counts)))>=0.9){yl <- ceiling(max(counts))+0.5
}else if((max(counts)-floor(max(counts)))>=0.4){yl <- ceiling(max(counts))
}else{yl <- floor(max(counts))+0.5}
if(yl<2.5){yl=2.5}
op<-barplot(counts1,width =colwidth,ylab=ylab,col=col,ylim=c(0,yl),space
=space,family=family)
axis(side=1,c(-100,op[c(-1,-dim(op)[1]),],100),tcl=-0.2, labels=FALSE)
#axis(side=1,c(-100,100),tcl=-0.2, labels=FALSE)
axis(side=2, c(-100,100),tcl=-0.2, labels=FALSE)
```

```
axis(side=3, c(-100,100),tcl=-0.2, labels=FALSE)
axis(side=4, c(-100,100),tcl=-0.2, labels=FALSE)
abline(h=-log10(0.05), col="blue",lty=2,lwd=1.5)
abline(h=-log10(0.01), col="red",lty=2,lwd=1.5)
text ( x=op,y=-yl/30,srt = srt, adj = 1,labels = labels1, xpd = TRUE, cex=cex,
family = family)
par(opar)
```

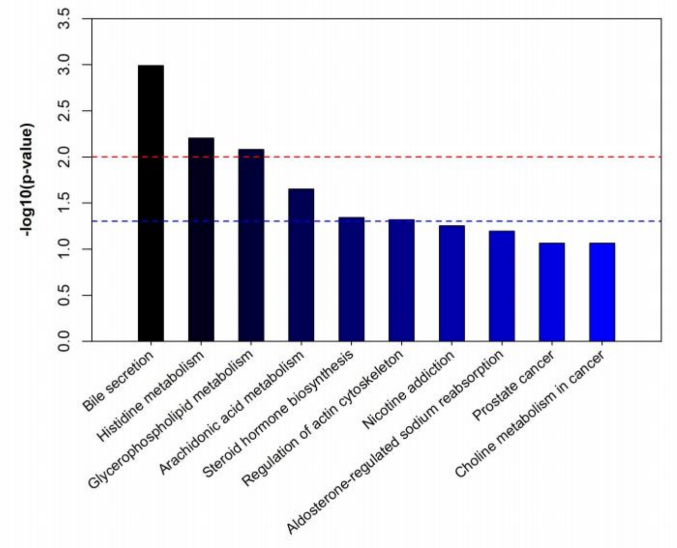

Heatmap:

```
library("pheatmap")
data1<- read.table("heatmap.txt",head = T,sep="/t",row.names=1,encoding="utf-
8",check.names=F,quote="",as.is=TRUE)
pheatmap(data1,treeheight_row=40,treeheight_col=40,
scale="row",cluster_cols=F,cluster_rows=T,
display_numbers=F,number_format="%.3f",
fontsize_ro=10,fontsize_col=15,cellwidth =18,
cellheight =10,show_colnames=TRUE,
color = colorRampPalette(c("green", "black","red"))(50000),
border_color="white")
```

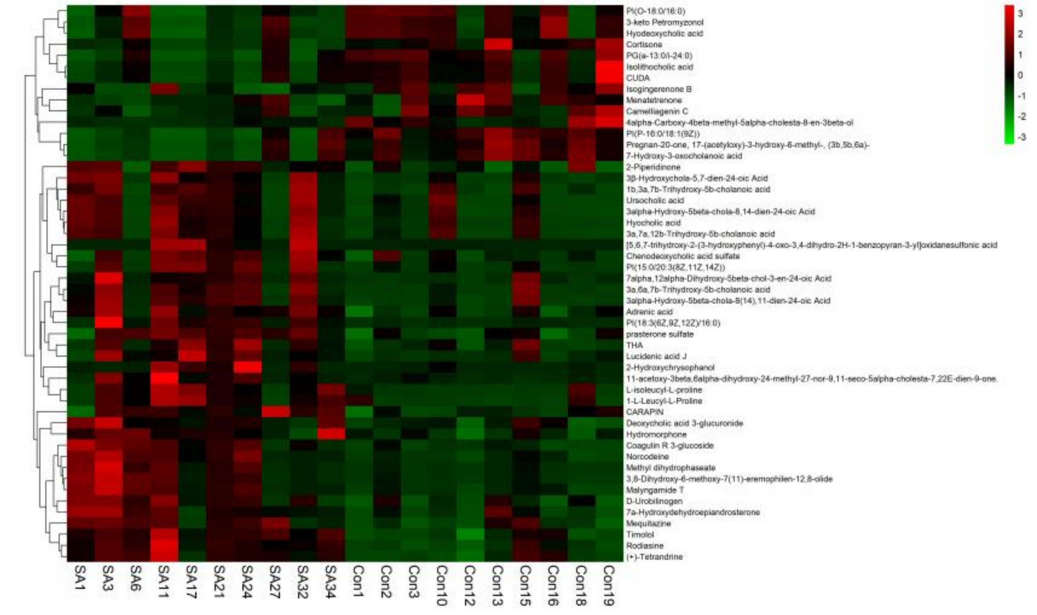

VIP score:

```
data<-openxlsx::read.xlsx(xlsxFile ="different metabolites.xlsx",sheet=1)
data1<-data[,c("Metabolites","VIP","average(SA)","average(Con)")]
names(data1)<-c("Metabolites","VIP","SA","Con")
data1<-data1[1:16,]
auto_vipmap<-function(data,
name=NA,
type=NA,
dpi=300,
compression ="zip",
width=15,
height =5,
order=T){
library("ggplot2",quietly = T)
library("patchwork",quietly = T)
data1<-data
names(data1)[1]<-"Metabolites"
names(data1)[2]<-"VIP"
if(order){data1<-data1[order(data1$VIP),]}
data1$Metabolites<-factor(data1$Metabolites,levels = data1$Metabolites)
data2<-data1[,c("Metabolites","VIP")]
plot1 <-ggplot(data2, aes(x = VIP, y = Metabolites)) +
geom_point() +
labs(y="",x="VIP Score")+
theme_bw()+
#scale_y_discrete(labels=NULL)+
theme(panel.background = element_blank(),
panel.grid=element_blank(),
axis.line = element_line(colour = "black"))+
```

```
theme(panel.grid.major.y = element_line(colour = "grey",linetype = 3))
data3<-data1[,-2]
data3[,"aver"]<-apply(data3[,-1], 1, mean)
data3[,2:(dim(data3)[2]-1)]<-data3[,2:(dim(data3)[2]-1)]/data3[,"aver"]
data3<-data3[,-dim(data3)[2]]
data3<-reshape2::melt(data3,id=c("Metabolites"))
names(data3)<-c("Metabolites","Group","value")
plot3 <-ggplot(data3, aes(x = Group,y=Metabolites,color=value)) +
geom_point(shape=15,size=7)+
labs(y="",x="")+
theme_bw()+
theme(panel.background = element_blank(),
panel.grid=element_blank(),
panel.border = element_blank(),
axis.line.x = element_line(colour = "black"))+
scale_y_discrete(name = NULL, labels = NULL, breaks = NULL)+
scale_colour_gradient(low="green",high="red",name="",breaks=c(min(data3$value),
max(data3$va
lue)*1.0),labels=c("low","high"))
plot <- {plot1 + plot3 + plot_layout(ncol = 2, widths = c(7, 1))}
if(is.na(name)){
return(plot)
}else{
if(type=="tiff"){
ggsave(name,plot,dpi=dpi,width=width,height=height,compression =compression)
}else{ggsave(name,plot,dpi=dpi,width =width,height = height)}
}
} a
uto_vipmap(data=data1,name="VIP-SA_Con.pdf",type="pdf")
auto_vipmap(data=data1,name="VIP-SA_Con.jpg",type="jpg")
```

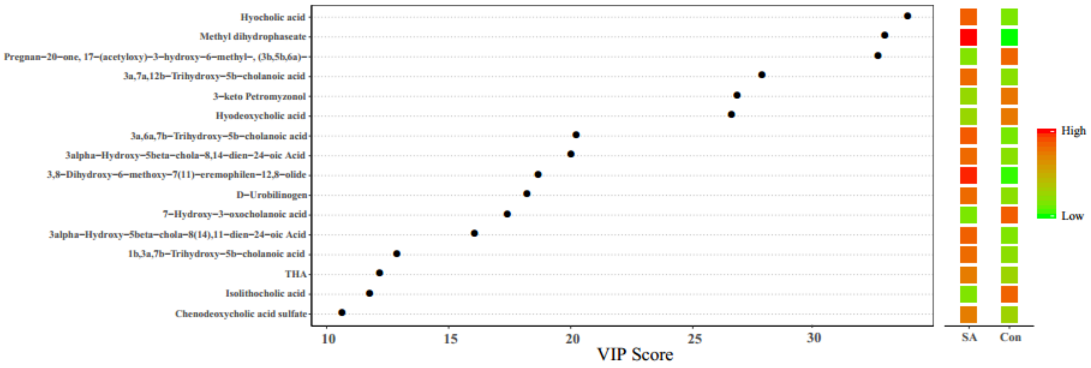

Correlation:

```
library(readxl)
library(tidyr)
setwd("~/Documents/R project/lessons/ ")
data1 <- read_excel("correlation analysis.xlsx",
                    sheet = "diversity and cytokines ") %>% as.data.frame()
data1<- data1[,-1]
multiCor<- function(data,x1,x2){

  b<- list()
  for (i in colnames(data)[1:x1]) {
    for (y in colnames(data)[(x1+1):x2]) {

      b[[i]][[y]]<-   cor.test(data[,i],data[,y],method = "pearson")

    }
  }
  d2<- sapply( names(b),function(x){
    sapply(names(b[[x]]), function(y){
      cbind( b[[x]][[y]][["estimate"]], b[[x]][[y]][["p.value"]])
    })}
  )
  d2<- d2 %>% as.data.frame()
  paste0(rep(colnames(data)[(x1+1):x2],each=2),c("_estimates","_pvalue"))
  d2$cor                                                                  <-
paste0(rep(colnames(data)[(x1+1):x2],each=2),c("_estimates","_pvalue"))
  return(d2)
}
d1<- multiCor(data1,9,21)
write.table(d1,"shee1.csv",sep = ",",col.names = T,row.names = F)
###################plot########################
data <- data1
if (!dir.exists("sheet1")) {
  (dir.create("sheet1"))
}
colnames(data)<- make.names(colnames(data))
if (!dir.exists("sheet1/x_y")) {
  (dir.create("sheet1/x_y"))
}
for (i in colnames(data)[1:9]) {
  for (y in colnames(data)[10:21]) {
    dir <-"sheet1/x_y/"
    pdf(file=paste0(dir,i,"_",y,".pdf"))
    plot(data[,i],data[,y], xlab =i, ylab = y)
    abline(lm(data[,y]~data[,i]), col = "blue")
```

```
        dev.off()
    }
}
if (!dir.exists("sheet1/y_x")) {
    (dir.create("sheet1/y_x"))
}
colnames(data)<- make.names(colnames(data))
for (i in colnames(data)[1:9]) {
    for (y in colnames(data)[10:21]) {
        dir <-"sheet1/y_x/"
        pdf(file=paste0(dir,y,"_",i,".pdf"))
        plot(data[,y],data[,i], xlab =y, ylab = i)
        abline(lm(data[,i]~data[,y]), col = "blue")
        dev.off()
    }
}
```

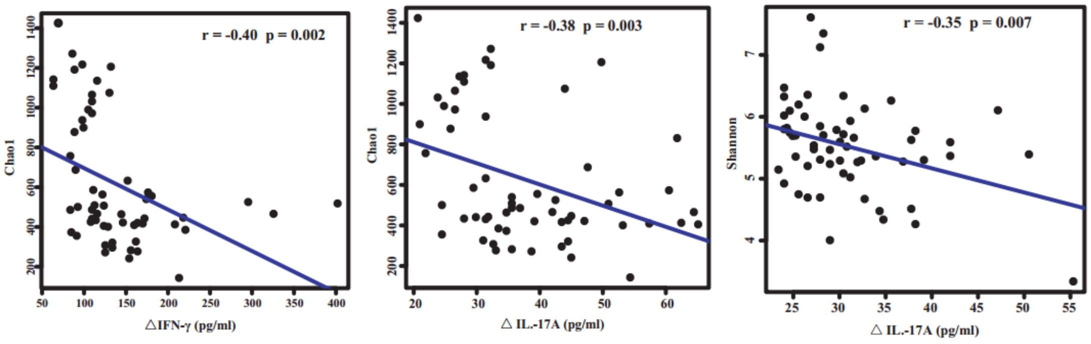

Network:

```
library("dplyr")
library("tidyr")
library("ggplot2")
library("scales")
library("network")
library("sna")
library("GGally")
library("igraph")
correlation<- read.csv("p0.05.xls",header=T,sep="\t")
colnames(correlation) <-
c("From","to","Correlation","Pvalue","Type","AdjPvalue","Significance","Positive_N
egati
ve")
category1 <- cbind(as.data.frame(unique(correlation$From)),"Metabolites")
category2 <- cbind(as.data.frame(unique(correlation$to)),"OTU")
colnames(category2) <- c("label","Category")
colnames(category1) <- c("label","Category")
nodes <- rbind(category1,category2)
```

```
em.cet<-as.character(nodes$Category)
names(em.cet) = nodes$label
edges<- correlation
em.net <- edges[, c("From", "to") ]
em.net <- network::network(em.net, directed = F)
em.net %v% "curr_empl_type" <-
em.cet[ network.vertex.names(em.net) ]
col<-ifelse(as.numeric(edges[,3])<0,"green","red")
network::set.edge.attribute(em.net, "n", abs(edges[, 3 ]) / 2)
network::set.edge.attribute(em.net, "m",col)
set.seed(10312016)
ggnet2(em.net, color = "curr_empl_type",size = 6,label=TRUE,label.size=1.0, palette
=
"Set2",arrow.size = 5, arrow.gap = 0.02,edge.alpha = 0.25, mode =
"fruchtermanreingold",edge.size="n",edge.color = "m",color.legend = "Category") +
theme(legend.position = "bottom")
```

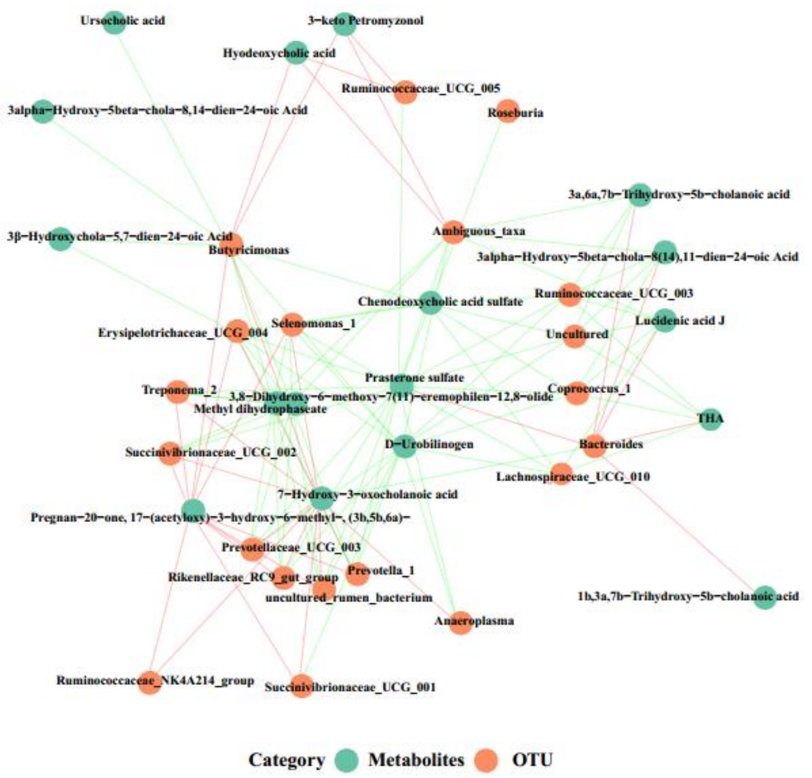

RDA:

```
library(vegan)
sp <- read.table(file=file.choose(),sep="\t",header=T,row.names=1)
sp
se <- read.table(file=file.choose(),sep="\t",header=T,row.names=1)
se
decorana(sp)
sp0 <- rda(sp ~ 1, se)
```

```
sp0
plot(sp0)
sp1 <- rda(sp ~ ., se)
sp1
plot(sp1)
new<-sp1$CCA
new
samples<-data.frame(sample=row.names(new$u),RDA1=new$u[,1],RDA2=new$u[,2
])
samples
species<-data.frame(spece=row.names(new$v),RDA1=new$v[,1],RDA2=new$v[,2])
species
envi<-data.frame(en=row.names(new$biplot),RDA1=new$biplot[,1],RDA2=new$bip
lot[,2])
envi
line_x = c(0,envi[1,2],0,envi[2,2],0,envi[3,2],0,envi[4,2],0,envi[5,2],0,envi[6,2])
line_x
line_y = c(0,envi[1,3],0,envi[2,3],0,envi[3,3],0,envi[4,3],0,envi[5,3],0,envi[6,3])
line_y
line_g = c("IL2"," IL17A"," IL17F","TNFa","IFNr")
line_g
line_data = data.frame(x=line_x,y=line_y,group=line_g)
line_data
library(ggplot2)
ggplot(data=samples,aes(RDA1,RDA2)) + geom_point(aes(color=sample),size=2) +
geom_point(data=species,aes(shape=spece),size=2) +
geom_text(data=envi,aes(label=en),color="blue") +
geom_hline(yintercept=0) + geom_vline(xintercept=0)+
  geom_line(data=line_data,aes(x=x,y=y,group=group),color="green") +
  theme_bw() + theme(panel.grid=element_blank())
ggsave("RDA2.PDF")
```

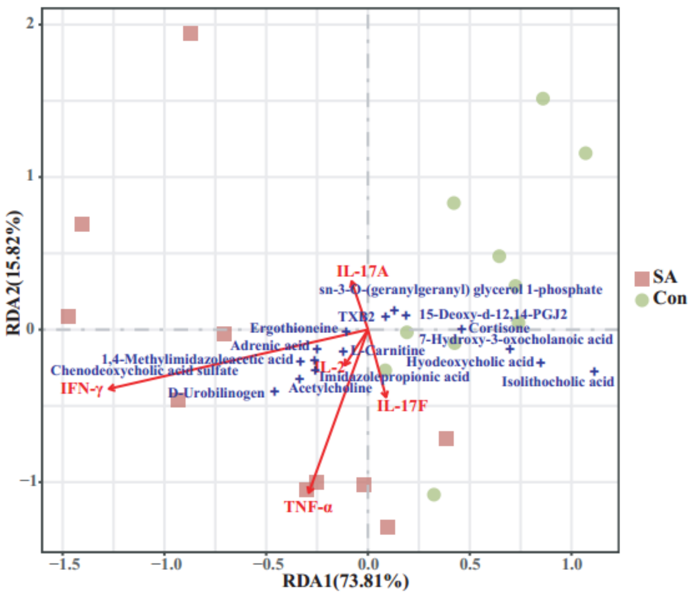

ROC：

```
data<-openxlsx::read.xlsx(xlsxFile ="heatmap.xlsx",sheet=1,rowNames=T)
data1<-data[1:2,]
data1<-as.data.frame(t(data1))
data2<-reshape2::melt(data1,id=c("Group"))
names(data2)<-c("Group","Metabolites","Expression")
library("ggplot2",quietly = T)
library("plotROC",quietly = T)
p<-ggplot(data2, aes(m = Expression, d = Group,color=Metabolites)) +
geom_roc(labels=F,n.cuts = 0)+
theme_bw()+
facet_wrap(~ Metabolites)+
theme(legend.position='none')+
xlab("1 - Specificity")+
ylab("Sensitivity")+
scale_x_continuous(expand = c(0.01, 0.01))+
scale_y_continuous(expand = c(0.01, 0.01))+
geom_hline(yintercept=c(0,1))+
geom_vline(xintercept=c(0,1))+
scale_colour_manual(values="black")+
geom_abline(slope=1, intercept=0,linetype="dashed")+
theme(text=element_text(family="sans"),
panel.grid=element_blank(),
plot.title = element_text(hjust = 0.5),
aspect.ratio=1,
axis.text=element_text(size=10),
axis.title=element_text(size=10),
legend.text=element_text(size=10),
```

```
legend.title=element_text(size=10),
plot.margin = unit(c(0.3,0.3,0.3,0.3),"in"))
ggsave("ROC.jpg",p,width=5,height=5)
ggsave("ROC.pdf",p,width=5,height=5)
```

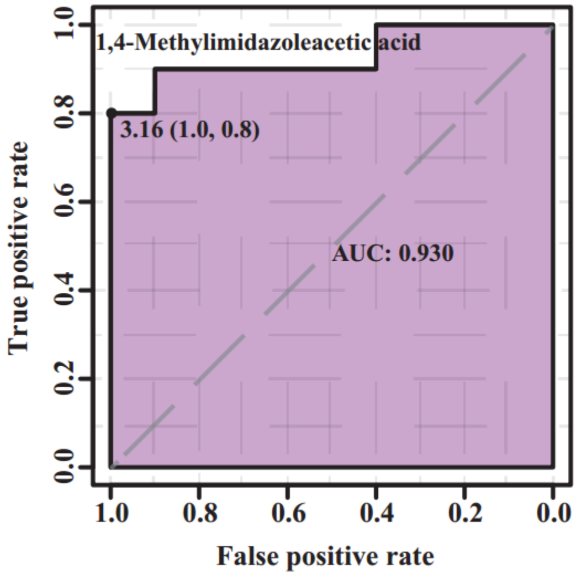

Supplement: Supplementary file 1 — Supplementary Information [file 41522_2021_199_MOESM1_ESM.pdf]
