# Supplementary material for: Interactions between gut microbiota and metabolites modulate cytokine network imbalances in women with unexplained miscarriage
Source: NPJ Biofilms Microbiomes. 2021 Mar 17;7:24. doi: 10.1038/s41522-021-00199-3 (PMC7969606; doi:10.1038/s41522-021-00199-3)
Supplement: Supplementary file 2 — Reporting Summary [file 41522_2021_199_MOESM2_ESM.pdf]

nature research

Corresponding author(s): Liping Feng

Last updated by author(s): Feb 5, 2021

Reporting Summary

Nature Research wishes to improve the reproducibility of the work that we publish. This form provides structure for consistency and transparency in reporting. For further information on Nature Research policies, see our Editorial Policies and the Editorial Policy Checklist.

Statistics

For all statistical analyses, confirm that the following items are present in the figure legend, table legend, main text, or Methods section.

n/a	Confirmed	
☐	☒	The exact sample size (*n*) for each experimental group/condition, given as a discrete number and unit of measurement
☐	☒	A statement on whether measurements were taken from distinct samples or whether the same sample was measured repeatedly
☐	☒	The statistical test(s) used AND whether they are one- or two-sided<br>*Only common tests should be described solely by name; describe more complex techniques in the Methods section.*
☐	☒	A description of all covariates tested
☐	☒	A description of any assumptions or corrections, such as tests of normality and adjustment for multiple comparisons
☐	☒	A full description of the statistical parameters including central tendency (e.g. means) or other basic estimates (e.g. regression coefficient) AND variation (e.g. standard deviation) or associated estimates of uncertainty (e.g. confidence intervals)
☐	☒	For null hypothesis testing, the test statistic (e.g. *F*, *t*, *r*) with confidence intervals, effect sizes, degrees of freedom and *P* value noted<br>*Give P values as exact values whenever suitable.*
☐	☒	For Bayesian analysis, information on the choice of priors and Markov chain Monte Carlo settings
☐	☒	For hierarchical and complex designs, identification of the appropriate level for tests and full reporting of outcomes
☐	☒	Estimates of effect sizes (e.g. Cohen's *d*, Pearson's *r*), indicating how they were calculated

Our web collection on statistics for biologists contains articles on many of the points above.

Software and code

Policy information about availability of computer code

Data collection	The raw data obtained from high-throughput sequencing were stored in FASTQ format. Low-quality sequences that had an average quality score below 20 were cut off using Trimmomatic software. After trimming, paired-end reads were assembled with FLASH software. The parameters of assembly were as follows: 10 bp of minimal overlapping, 200 bp of maximum overlapping and a 20% maximum mismatch rate. Further de-noising including the removing of ambiguous base (N), sequences below 200 bp, and chimeras was performed with QIIME software (version 1.8.0). The obtained clean reads were then subjected to primer-sequence removal and clustering to generate operational taxonomic units (OTUs) with a 97% similarity cutoff using Vsearch software. The most abundant one was selected as the representative read of each OTU. All representative reads were annotated and blasted against the Silva database (v. 123) using RDP classifier.
Data analysis	A full record of all statistical analysis and R scripts used in this paper are included in Supplementary File 2.

For manuscripts utilizing custom algorithms or software that are central to the research but not yet described in published literature, software must be made available to editors and reviewers. We strongly encourage code deposition in a community repository (e.g. GitHub). See the Nature Research guidelines for submitting code & software for further information.

Data

Policy information about availability of data

All manuscripts must include a data availability statement. This statement should provide the following information, where applicable:

- Accession codes, unique identifiers, or web links for publicly available datasets
- A list of figures that have associated raw data
- A description of any restrictions on data availability

Sequencing data, metabolites profiles, and metadata for all samples used in this study have been deposited in Figshare (http://dx.doi.org/10.6084/m9.figshare.

12254363; 12263678; 12263705， respectively). A patient metadata file, which includes the time of sample collection and following up of the last pregnancies, was included in Supplementary File 1. A full record of all statistical analysis and R scripts used in this paper are included in Supplementary File 2.

Field-specific reporting

Please select the one below that is the best fit for your research. If you are not sure, read the appropriate sections before making your selection.

☒ Life sciences ☐ Behavioural & social sciences ☐ Ecological, evolutionary & environmental sciences

For a reference copy of the document with all sections, see nature.com/documents/nr-reporting-summary-flat.pdf

Life sciences study design

All studies must disclose on these points even when the disclosure is negative.

Sample size	G*POWER was used for statistical power analyses for microbiome and cytokines analysis in this study. The type of a priori power analysis was performed with the following parameters: two tails, effect size at 0.8, α err prob at 0.05, power at 0.8, and allocation ratio N2/N1 at 0.5. The calculated sample size for group 1(case group) is 39, and for group 2 (control group) is 19. Our sample size is comparable to other published studies.
Data exclusions	Participants who have taken antibiotics, probiotics, prebiotics, or synbiotics two months prior to sample collection were excluded.
Replication	We confirm the replication was successful.
Randomization	This ia a case-control study. Women in the first trimester of pregnancy with an unexplained miscarriage or that underwent elected abortion were recruited.
Blinding	This is a case-control study.

Reporting for specific materials, systems and methods

We require information from authors about some types of materials, experimental systems and methods used in many studies. Here, indicate whether each material, system or method listed is relevant to your study. If you are not sure if a list item applies to your research, read the appropriate section before selecting a response.

Materials & experimental systems

n/a	Involved in the study	
☒	☐	Antibodies
☒	☐	Eukaryotic cell lines
☒	☐	Palaeontology and archaeology
☒	☐	Animals and other organisms
☐	☒	Human research participants
☒	☐	Clinical data
☒	☐	Dual use research of concern

Methods

n/a	Involved in the study	
☒	☐	ChIP-seq
☐	☒	Flow cytometry
☒	☐	MRI-based neuroimaging

Human research participants

Policy information about studies involving human research participants

Population characteristics	Eligible cases were women (i) who were less than 35 years of age with a pre-pregnancy body mass index (BMI) of 18.5-23.9 kg/m2 (normal reference value standards of BMI for Chinese); (ii) who had no successful pregnancies (their miscarriages occurred before 14 weeks of gestation); and (iii) whose current male partner had normal semen testing (sperm density > 15 million/mL, and sperm motility > 60%) by computer-assisted analysis. Age and gestational week-matched controls were women with normal early pregnancy who elected to have an abortion.
Recruitment	Eligible cases were women (i) who were less than 35 years of age with a pre-pregnancy body mass index (BMI) of 18.5-23.9 kg/m2 (normal reference value standards of BMI for Chinese); (ii) who had no successful pregnancies (their miscarriages occurred before 14 weeks of gestation); and (iii) whose current male partner had normal semen testing (sperm density > 15 million/mL, and sperm motility > 60%) by computer-assisted analysis. Age and gestational week-matched controls were women with normal early pregnancy who elected to have an abortion. Subjects with high-risk factors of miscarriage were excluded.
Ethics oversight	The Ethics Committees of Xinhua Hospital affiliated to Shanghai Jiao Tong University School of Medicine

Note that full information on the approval of the study protocol must also be provided in the manuscript.

Flow Cytometry

Plots

Confirm that:

☒ The axis labels state the marker and fluorochrome used (e.g. CD4-FITC).

☒ The axis scales are clearly visible. Include numbers along axes only for bottom left plot of group (a 'group' is an analysis of identical markers).

☒ All plots are contour plots with outliers or pseudocolor plots.

☒ A numerical value for number of cells or percentage (with statistics) is provided.

Methodology

Sample preparation	The peripheral blood was collected and centrifuged for 20 min at 1,000 × g to collect serum. Serum samples were diluted two folds with Assay Buffer before being tested.
Instrument	Cytomics FC 500 of Beckman Coulter company.
Software	LEGENDplex 8.0 software from BioLegend.
Cell population abundance	The LEGENDplex Th Cytokine Panel is a bead-based multiplex assay panel, using fluorescence-encoded beads suitable for use on various flow cytometers. Beads are differentiated by size and internal fluorescence intensities. Each bead set is conjugated with a specific antibody on its surface and serves as the capture beads for that particular analyte. When a selected panel of capture beads is mixed and incubated with a sample containing target analytes specific to the capture antibodies, each analyte will bind to its specific capture beads. After washing, a biotinylated detection antibody cocktail is added, and each detection antibody in the cocktail will bind to its specific analyte bound on the capture beads, thus forming capture bead-analyte-detection antibody sandwiches. Streptavidin-phycoerythrin (SA-PE) is subsequently added, which will bind to the biotinylated detection antibodies, providing fluorescent signal intensities in proportion to the amount of bound analytes. The concentration of a particular analyte is determined using a standard curve generated in the same assay. This panel allows simultaneous quantification of 13 human cytokines, including IL-2, 4, 5, 6, 9, 10, 13, 17A, 17F, 21, 22, IFN-γ and TNF-α.
Gating strategy	The Human Th Cytokine Panel uses two sets of beads. Each set has a unique size that can be identified based on their forward scatter (FSC) and side scatter (SSC) profiles (Beads A and Beads B). Each bead set can be further resolved based on their internal fluorescence intensities. The internal dye can be detected using FL3, FL4, OR APC channel, depending on the type of flow cytometer used. The smaller Beads A consists of 6 bead populations and the larger Beads B consists of 7 bead populations. Using a total of 13 bead population distinguished by size and internal fluorescent dye, the Human Panel allows simultaneous detection of 13 cytokines in a single sample. Each analyte is associated with a particular based sets as indicated.

☒ Tick this box to confirm that a figure exemplifying the gating strategy is provided in the Supplementary Information.
